# Linking synthesis and structure descriptors from a large collection of synthetic records of zeolite materials

**DOI:** 10.1038/s41467-019-12394-0

**Published:** 2019-10-01

**Authors:** Koki Muraoka, Yuki Sada, Daiki Miyazaki, Watcharop Chaikittisilp, Tatsuya Okubo

**Affiliations:** 1 Department of Chemical System Engineering, The University of Tokyo, 7-3-1 Hongo, Bunkyo-ku, Tokyo, 113-8656 Japan; 2 Present Address: Research and Services Division of Materials Data and Integrated System (MaDIS), National Institute for Materials Science (NIMS), 1-1 Namiki, Tsukuba Ibaraki, 305-0044 Japan

**Keywords:** Inorganic chemistry, Materials chemistry, Porous materials

## Abstract

Correlating synthesis conditions and their consequences is a significant challenge, particularly for materials formed as metastable phases via kinetically controlled pathways, such as zeolites, owing to a lack of descriptors that effectively illustrate the synthesis protocols and their corresponding results. This study analyzes the synthetic records of zeolites compiled from the literature using machine learning techniques to rationalize physicochemical, structural, and heuristic insights to their chemistry. The synthesis descriptors extracted from the machine learning models are used to identify structure descriptors with the appropriate importance. A similarity network of crystal structures based on the structure descriptors shows the formation of communities populated by synthetically similar materials, including those outside the dataset. Crossover experiments based on previously overlooked structural similarities reveal the synthesis similarity of zeolites, confirming the synthesis–structure relationship. This approach is applicable to any system to rationalize empirical knowledge, populate synthesis records, and discover novel materials.

## Abstract (web summary)

Understanding zeolite synthesis-structure relationships remains challenging owing to the number of variables involved in their preparation. Here the authors analyze zeolite synthetic records from the literature via machine learning and find communities of synthetically related materials with previously overlooked similarities.

## Introduction

Driven by the increased computational power, the advances in algorithms development, and the availability of a massive amount of data, applications of machine learning have expanded to solve human-level problems^1–3^, including those in materials science^4–6^. The datasets in materials science casted to the machine learning are heavily derived from theoretical calculations^7–11^. Once trained, the machine learning can be applied to high-throughput screening of thousands or even millions of material candidates. These exhaustive in silico data-mining approaches enable us to identify the remarkable materials from large, computationally generated database^12–15^. As a result, the central research question is returning to the conventional one: how to synthesize the targeted new materials?

Synthesis of materials can also receive the benefit from machine learning. For example, a series of supervised classification models was constructed from a large collection of experimental data to predict synthetic consequences using a set of synthesis descriptors^16,17^. This machine learning-based approach to the experimental database enables us to extract the most significant synthesis descriptors from chemical space with a high dimension and massive entries, which is sometimes very hard to be handled by humans. In particular, the pattern recognition capability of machine learning is thought to be exceptionally effective for the materials that are synthesized through kinetically controlled pathways, which are difficult to be treated by straightforward methodologies.

This holds for zeolites, a class of microporous aluminosilicate crystals^18^. It is generally accepted that zeolites are formed as metastable phases via kinetically controlled pathways^18–20^. Zeolites having different crystalline phases can be obtained by only slight change of the synthesis descriptors, such as chemical compositions of raw materials, heating time, heating temperature, and types of organic molecules called organic structure-directing agents (OSDAs)^21,22^. Consequently, it is hardly possible to describe the complex energy landscape to identify the crystalline phases of the zeolite products for a given set of synthesis descriptors by theoretical calculations and experiments.

**Fig. 1:**
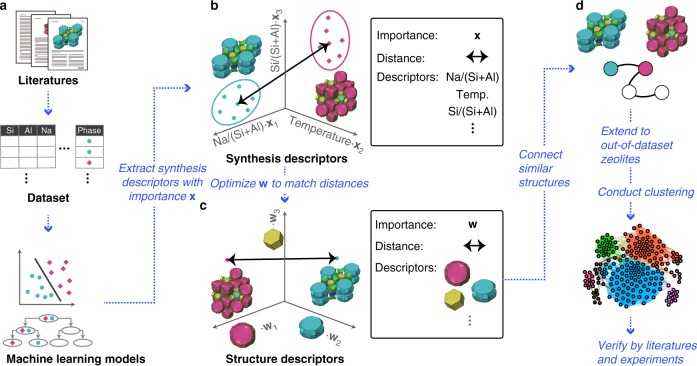
Workflow to link synthesis descriptors to structure descriptors in zeolites. **a** Machine learning models were constructed from experimental records in the literature; the dataset contains synthesis descriptors and corresponding outcomes. **b** Synthesis descriptors extracted from the machine learning models mapped the synthesizable domains of zeolites onto a multidimensional (kinetic) phase diagram. The weight, **x**_*i*_, indicates the importance of each synthesis descriptor, *i*, obtained from the machine learning models. The synthesis similarity is represented by the distance between the centers of the synthesis conditions for each phase. **c** Structure descriptors define the structural similarity in a multidimensional space representing the presence or absence of building units. To link the synthesis descriptors to the structure descriptors quantitatively, the weight, **w**_*j*_, for each structure descriptor, *j*, was optimized to yield the structural similarity (arrow in **c**) close to the synthesis similarity (arrow in **b**). **d** A network was constructed by connecting structurally similar zeolites based on the structure descriptors. The resulting clustering was verified with historical data and our experiments

Despite the long history of zeolite synthesis^18,19^, the causal relationship between synthesis descriptors and the resulting zeolite products remains unclear. As shown in Fig. 1b, the phase change between zeolites is often dominated by multiple synthesis descriptors, making the drawing of boundaries on two-dimensional kinetic phase diagrams difficult^22^. Even when focusing on a single synthesis descriptor, other factors can be changed through the solution chemistry^23,24^; therefore, general relationships between structure descriptors and synthesis descriptors are difficult to elucidate^22^. Another difficulty arises in the extraction of structure descriptors. One of the common strategies to develop the structure descriptors is to decompose the chemical topology into a collection of building units^25^. In the case of metal-organic frameworks (MOFs), it is relatively simple because MOFs are constructed from distinct organic linkers and inorganic units^26^. On the other hand, the frameworks of zeolites are built solely from a collection of TO_4/2_ (T is tetrahedral atoms such as Si and Al) primary building units, making the identification of structure descriptors inconclusive. Nevertheless, several definitions of secondary building units^27–29^ have been proposed by focusing on the common motifs observed in different zeolite structures, such as those shown in Fig. 1c and Supplementary Fig. 1. The correlations between the structure similarity and the synthesis conditions have been observed in several cases^30–32^, though the analyses of precursor species suggest that the building units are not necessarily present in the intermediate mixtures^33,34^.

To correlate synthesis descriptors and structure descriptors, a series of experimental data (Supplementary Table 1) is compiled with several synthesis descriptors covering a wide range of the chemical space in the OSDA-free synthesis of aluminosilicate zeolites (Fig. 1a). The resulting dataset contains 686 synthesis conditions. The products include 22 crystalline phases (Supplementary Fig. 2) and an amorphous solid. The pattern recognition capability of machine learning algorithms is used to rationalize the empirical and physicochemical knowledge behind the large number of experimental records. Further, graph theory is employed to identify structural similarities in zeolite structures, reflecting similarity in the synthesis by clustering synthetically similar zeolites based on similarities in the structure descriptors (Fig. 1d). Crossover experiments between structurally related materials reveal previously overlooked synthesis similarities, demonstrating the broad applicability of the synthesis–structure relationship.

## Results

### Construction of machine learning models

To link the synthesis descriptors and structure descriptors, it is necessary to focus on the primary descriptors that are closely related to synthetic consequences^35^. This problem can be formulated to find the importance of the synthesis descriptors (**x**) and the structure descriptors (**w**) in Fig. 1, in which **x** is the weight that effectively separates two different domains in the weighted chemical space, while **w** is calculated to have the proper weight to reproduce the similarity (or distance) between zeolite structures in the weighted chemical space (Fig. 1).

Chemical compositions, which are the most significant synthesis descriptor for zeolite phase selections^22^, are typically expressed as molar ratios relative to one or more chemical components. To find the most appropriate chemical component by which the other components are to be divided (i.e., the denominator), various machine learning models were trained to predict the synthesis results from synthesis descriptors including temperature, heating time, and chemical composition with different standard denominators. As summarized in Supplementary Table 2, the extreme gradient boosting (XGBoost) and random forest models outperformed the other models, with test accuracies of 75–80%. Among the best combinations, the XGBoost model with (Si + Al) as the standard denominator was selected because its hyperparameter tuning is computationally efficient and (Si + Al) represents the total amount of tetrahedral atoms in the synthesis system.

In addition to chemical compositions, heating temperatures, and heating times, aging conditions^30^ and sources of reactants^36^ have been known to highly affect the zeolite synthesis. We encoded these variables into one-hot vectors and added to the synthesis descriptors for the construction of machine learning models. As shown in Supplementary Table 3, additional descriptors did not improve the test accuracy, except that the application of the random forest on all synthesis descriptors showed 82% accuracy. Considering the little improvement and the lack of detailed conditions in early literature^22^, we decided to exclude the one-hot vectors. Although this is beyond the scope of this research, our developed machine learning models based on XGBoost can predict not only synthesis results but also the probability associated with them as it can be used to quantify the likeliness of the formation of specific zeolite in a given synthesis condition.

**Fig. 2:**
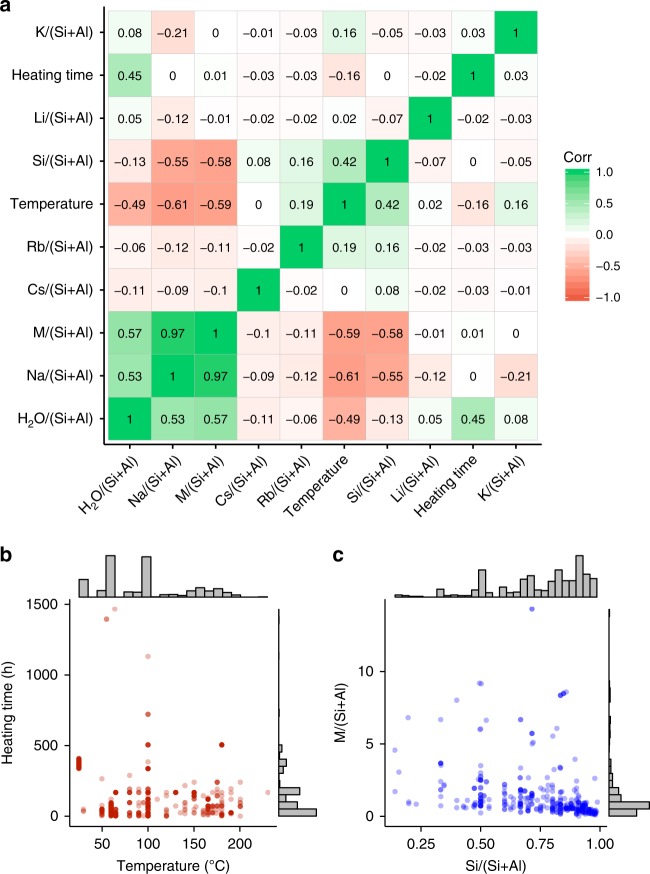
Overview of the dataset. **a** Correlogram showing the relations between synthesis descriptors of all synthesis conditions that crystallize zeolites. Distribution of the dataset showing **b** heating time versus temperature and **c** M/(Si + Al) versus Si/(Si + Al). A total amount of cations, M/(Si + Al), is calculated with charge consideration (i.e., (M^+^ + 0.5M^2+^)/(Si + Al))

Not all attempts to crystallize zeolites are successful. Improper heating conditions and/or chemical compositions can produce amorphous aluminosilicates. To examine the relationships between the synthesis descriptors within the synthetic ranges that crystallize zeolites, we calculated the correlations as shown in Fig. 2a. Positive or negative correlations signify a pair of synthesis descriptors that is mutually dependent in the applicable domain for synthesis of zeolites. Positive correlation indicates that paired descriptors typically change in the same direction (either increase or decrease) to successfully crystallize zeolites, while negative value means descriptors change oppositely.

The strongest correlation was observed between Na/(Si + Al) and M/(Si + Al), suggesting that the most frequently used cation is Na probably due to its ability to crystallize a variety of zeolite structures. Other sources of alkali metal cations including Li/(Si + Al) and K/(Si + Al) showed very weak negative correlation, confirming the importance of Na in the dataset. Relatively strong correlations were observed between M/(Si + Al) versus temperature, Si/(Si + Al), and H_2_O/(Si + Al). The negative correlation between M/(Si + Al) and temperature is reasonable considering that the increase of one of them generally enhances the kinetics of synthesis. The conditions with too high alkalinity and too high temperatures are expected to be beyond the appropriate domain of chemical space for crystallization of zeolites, while those with too low alkalinity and too low temperatures are sometimes not sufficient to foster the dissolution and polymerization of reactants and intermediates, respectively.

The negatively correlated relation between M/(Si + Al) and Si/(Si + Al) can be described by the solubility of Al sources. In typical conditions, Al sources tend to exist in the solid or gel phase^22,30^ throughout the synthesis due to its poor solubility in alkaline aqueous media. Therefore, the balance between the amounts of Al and M must be critical because Al sources must be dissolved, at least partially, to be involved in the reactions forming aluminosilicates, and the alkalinity has to be not too high to allow the formation of the crystallized products. The positive correlation between M/(Si + Al) and H_2_O/(Si + Al) suggests that the amount of hydroxide relative to the amount of water must be considered, indicating the effects of solution chemistry of silicates and aluminates in the crystallization of zeolites. As remarked here, chemically reasonable insights can be obtained from the general correlations among synthesis descriptors.

We also mapped the dataset by selecting sets of the synthesis descriptors as shown in Fig. 2b and c. In the dataset, synthesis of zeolites covered a wide range of temperatures from ambient temperature to 230 °C (Fig. 2b). In the lower temperature range, the most frequent temperatures were ambient temperatures, 60 °C, and 100 °C, while at higher temperatures the distribution of data was relatively uniform. The fastest synthesis in the dataset was the crystallization of **LTA** at 200 °C for 30 min^37^, while the longest synthesis took more than 2 months with relatively low temperature of 64 °C^38^, suggesting the diverse time scale in the dataset. Besides these outliers, most of the syntheses were carried out within 3 weeks as can be seen in the distribution of heating time (Fig. 2b). The negative correlation between M/(Si + Al) and Si/(Si + Al) is confirmed in Fig. 2c. The plot revealed that the majority of the zeolite synthesis was done with the range of Si/(Si + Al) > 0.5 and M/(Si + Al) < 3. Distribution of the dataset on these synthesis descriptors for each crystalline phase is shown in Supplementary Fig. 3.

### Interpretation of the model and thermodynamic insights

**Fig. 3:**
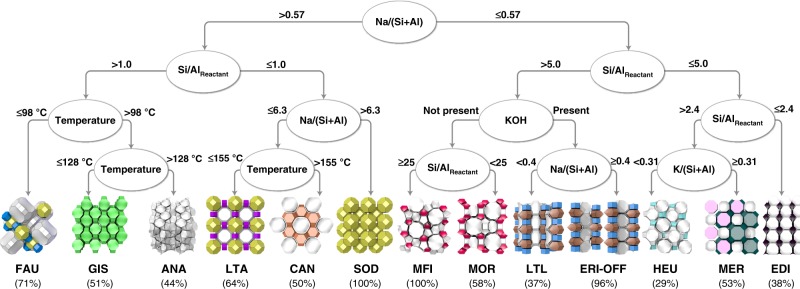
Decision tree constructed from the trained model with the highest accuracy of the XGBoost. In OSDA-free synthesis of zeolites, the most significant synthesis descriptors for zeolite phase selection are the amounts of SiO_2_, Al_2_O_3_, MOH (M = Li, Na, K, etc.), and H_2_O present in the synthesis mixture. Machine learning models including XGBoost, support vector machine, decision tree, and random forest were trained to predict synthesis results from synthesis descriptors including temperature, heating time, and chemical compositions with different standard denominators. The trained model with the highest accuracy was the XGBoost model using (Si + Al) as the denominator, and this model was interpreted as a decision tree shown here with a depth of 4. The complete tree (depth = 12) can be found in Supplementary Figs. 5–11. The most dominant crystalline phases in the predictions are presented. The percentages represent the fractions that the dominant phases appear in the deeper branches in the complete tree

Machine learning models such as XGBoost and random forest can be difficult to interpret because they are composed of multiple classifiers. One approach for interpreting these black box models is to derive the importance of the descriptors. The importance of the synthesis descriptors calculated from the XGBoost model was high for Si/(Si + Al), Na/(Si + Al), heating time, and H_2_O/(Si + Al) (Supplementary Fig. 4). Another interpretation approach is the application of interpretable models including decision trees for trained models^16^. The XGBoost model with the best performance (test accuracy = 80%) was interpreted as the decision tree (test accuracy = 76%) shown in Fig. 3. The syntheses were first divided based on the Na/(Si + Al) ratio. Zeolite structures obtained with high Na/(Si + Al) included **FAU**, **LTA**, and **SOD**, while lower Na/(Si + Al) mixtures preferred the formation of structures such as **MFI**, **MOR**, and **LTL**. The next boundary for the high Na/(Si + Al) groups was defined at the Si/(Si + Al) ratio of 0.5, which corresponds to the Si/Al ratio in the synthesis mixtures of 1 (note that Si/(Si + Al) was the actual synthesis descriptor used in the machine learning models, but to simplify the discussion Si/Al_Reactant_ is used hereafter, as this is the value typically described in the literature). This is interesting because the lowest Si/Al in solid zeolite products (Si/Al_Product_) is also 1 owing to Löwenstein’s rule^39^, which forbids the formation of Al–O–Al bonds. As a result, the chemistry of Si-rich and Al-rich conditions is substantially different. The fact that the machine learning model built solely from experimental data can acquire such chemically reasonable knowledge proves the effectiveness of the method used here.

**Fig. 4:**
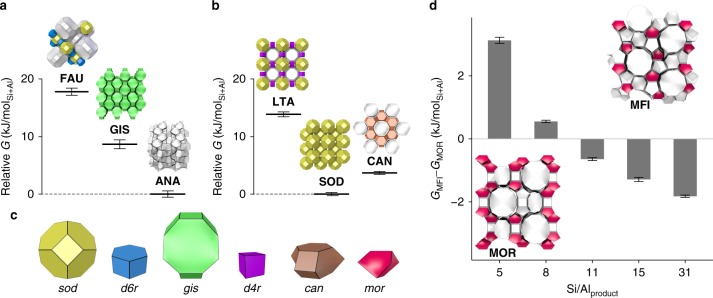
Relative Gibbs free energies of zeolites and the structural similarity between the crystal structures. **a** The relative Gibbs free energies of zeolites with Si/Al_Product_ = 2. **b** The relative Gibbs free energies of zeolites with Si/Al_Product_ = 1 estimated based on the Metropolis Monte Carlo simulations. **c** Representative building units found in **FAU**, **GIS**, **ANA**, **LTA**, **SOD**, **CAN**, **MFI**, and **MOR** structures. **d** The Gibbs free energy of **MFI** relative to **MOR** at different Si/Al_Product_. Error bars indicate standard deviation in five independent simulations

The three major phases observed in the branches with Si/Al_Reactant_ > 1.0 were **FAU**, **GIS**, and **ANA**, which were separated by the synthesis temperature. **FAU** was the most dominant phase at the lowest temperature, while **ANA** is dominated at the highest temperature. This is in line with the phase change from **FAU** to **GIS** to **ANA** described based on Ostwald’s step rule^40^—a commonly observed phenomenon in crystallization processes, in which multiple metastable phases are formed sequentially until reaching a stable phase^20,41^. Owing to the difficulty in direct evaluation of thermodynamic properties of zeolites, a previous study^40^ estimated the thermodynamic stability of different zeolites by comparing the density of zeolites in their pure-silica compositions and correlating it with their enthalpy of formation^20^. This kind of interpretation, however, has to be taken very carefully because (i) the thermodynamic properties and density of zeolites depend on the compositions and atomic configurations^42^, (ii) the calorimetric relationship between transition enthalpy and density is rather qualitative^20^, and (iii) the thermodynamic stability should be quantified by the Gibbs free energy rather than enthalpy^20,41^. Instead of using the density as the descriptor of the thermodynamic stability, the Metropolis Monte Carlo method^43^ was employed to estimate the Gibbs free energies by considering the effects of the composition and atomic configuration (see computational details in the section “Methods”). The Gibbs free energies of zeolites with Si/Al_Product_ = 2 depicted in Fig. 4a are consistent with Ostwald’s step rule, exhibiting the **FAU**-to-**GIS**-to-**ANA** transformation from lower to higher densities^40^. **FAU** is the least stable structure that progressively transforms to **GIS**, and finally **ANA**.

Ostwald’s step rule was also used previously to explain the **LTA**-to-**SOD**-to-**CAN** transformation by elevating heating temperature and/or extending heating time^40^. The temperature dependence of the **LTA**-to-**CAN** transformation was described in the decision tree, whereas **SOD** was separated based on Na/(Si + Al). According to the Gibbs free energies for Si/Al_Product_ = 1 (Fig. 4b), **LTA** exhibited a higher energy than **SOD** and **CAN**, implying the formation of **LTA** in the early stage of crystallization according to Ostwald’s step rule. The Gibbs free energy of **CAN**, however, was higher than that of **SOD**, contradicting the previous discussion based on their densities^40^. These results suggest that the **LTA**-to-**SOD** and **LTA**-to-**CAN** transformations proceeded according to Ostwald’s step rule, while **SOD**-to-**CAN** may not. Compared to the wide synthetic range yielding the **FAU**-to-**GIS**-to-**ANA** transformation, the range of phase transformations in Al-rich conditions seems to be narrower^40^. Especially, the **SOD**-to-**CAN** transformation typically involves incomplete crystallization and/or impurity^40,44^, suggesting a limited applicability of the **SOD**-to-**CAN** transformation. It is noteworthy that our calculations did not consider water that could have major impact on the stability of Al-rich zeolites, which should be taken into account for further studies.

The right side of the decision tree in Fig. 3 satisfied Na/(Si + Al) ≤ 0.57. As discussed above, the Al/(Si + Al) and M/(Si + Al) ratios were positively correlated in the chemical space that can yield zeolite, implying the smaller amount of Na/(Si + Al) requires the reduction of Al/(Si + Al) for the successful crystallization. As expected, the right side of the decision tree involved the conditions with higher Si/Al_Reactant_. Akin to the left side, the second boundary employed Si/Al_Reactant_ as the descriptor, again confirming the importance of Na/(Si + Al) and Si/(Si + Al) (Supplementary Fig. 4). **HEU** and **MER** were obtained as the major products at 2.4 < Si/Al_Reactant_ ≤ 5.0, depending on K/(Si + Al). At Na/(Si + Al) ≤ 0.57 and Si/Al_Reactant_ ≤ 2.4, **EDI** was the dominant phase. Note that in this branch other metal cations, such as Li and Tl are required to crystallize zeolites partly due to the insufficient amount of Na.

At Na/(Si + Al) ≤ 0.57 and Si/Al_Reactant_ > 5.0, **MFI** or **MOR** were obtained as the major products in the absence of K (Fig. 3). According to the empirical knowledge, conditions with high Si/Al_Reactant_ and low alkalinity favor the formation of zeolites containing five-membered ring units (*5r*, see Supplementary Fig. 1)^31,42^. The extraction of such empirical knowledge without providing any structural or topological information validates our approach. The boundary between **MFI** and **MOR** was drawn at Si/Al_Reactant_ = 25, which is consistent with previous reports, where high-silica conditions favored **MFI** while low-silica conditions tended to produce **MOR** in OSDA-free conditions^31,42,45^. We tried to rationalize this phase boundary by calculating the Gibbs free energy at different Si/Al_Product_ (Fig. 4d). The results suggest the thermodynamic stability of **MFI** over **MOR** at higher Si/Al_Product_, which is in accordance with the higher density of **MFI** under a pure-silica composition^45^. However, when Al and Na increase, **MOR** is stabilized. This transition occurred at a Si/Al_Product_ of 8–11, which is highly consistent with the experimental results^31,45^. Although synthesis using zeolites as reactants is out of scope for the current dataset (see the “Methods” section), a very recent report on **MFI**-to-**MOR** transformation starting from **FAU** as the reactant is remarkable^46^. As is commonly observed in seed-directed, OSDA-free synthesis of zeolites, Si/Al decreases upon progress of reaction^31^. In the recent report^46^, Si/Al_Reactant_ = 31 decreased to Si/Al_Product_ = 16 (**MFI**), and then Si/Al_Product_ = 6 (**MOR**), which is consistent with the relationship between structure versus composition in Fig. 4d, again indicating the reliability of our computational method. It is noteworthy that this recent report also suggested the limitation of zeolite density to predict Ostwald’s step rule for certain zeolite transformations (vide supra).

When K was present at relatively high Si conditions (Si/Al_Reactant_ > 5.0), **LTL** or **ERI**–**OFF** were predominant (Fig. 3). The increased alkalinity derived from the Na and K can dissolve a much greater amount of silicates, thereby yielding a lower Si/Al_Product_. As a result, zeolite structures without *5r* units, such as **LTL** and **ERI**–**OFF**, can be obtained (see also Supplementary Fig. 12). **LTL**, **ERI**, and **OFF** are structurally similar because they share *d6r* and *can* units (Supplementary Fig. 12a). Interestingly, structural similarity was also observed in the neighboring branch, where **MFI** and **MOR** share *mor* units (Fig. 4c, d). Such structural similarity has been used as a guideline in seed-directed zeolite syntheses^31^. Supplementary Table 4 lists the chemical compositions of the reactants in seed-directed, OSDA-free synthesis of zeolites, in which the zeolite products obtained with and without seed crystals are different but contain common building units. When these conditions are applied to the decision tree in Fig. 3, interestingly, all of the seed-directed syntheses containing this structural similarity fall on the branches of **MFI**, **MOR**, **LTL**, or **ERI**–**OFF**. It should be noted that these seed-directed syntheses were not used to train the machine learning models. Under these conditions of Na/(Si + Al) ≤ 0.57 and Si/Al_Reactant_ > 5.0, the structural similarity may be more pronounced in determining the zeolite products.

We further analyzed the possible Al distributions in the *mor* and *d6r* units. As is known, in addition to the Al–O–Al bond^39^, the Al–O–Si–O–Al sequence is not likely to be present in both units because they can energetically destabilize the zeolite structures, which is called as Löwenstein’s rule and Dempsey’s rule, respectively^47–49^. All possible configurations of Al in the *mor* and *d6r* units when Al was introduced as much as possible while avoiding the formation of Al–O–Al and Al–O–Si–O–Al bonds are present in Supplementary Fig. 13. In both units, the average Si/Al of these atomic configurations were 5, identical to the Si/Al_Reactant_ in the decision boundary. At Si/Al_Reactant_ > 5.0, the *mor* and *d6r* can be formed without forming Al–O–Al and Al–O–Si–O–Al bonds, while these unstable atomic sequences are inevitable at Si/Al_Reactant_ < 5.0. Although the actual Al distribution is not random but biased^48,50,51^, the topological characteristics inherent in the *mor* and *d6r* do not seem to be unconnected to the decision boundary at Si/Al_Reactant_ = 5.0.

We hypothesize that conditions with Na/(Si + Al) > 0.57 are too harsh for survival of certain crucial precursors, which can be aluminosilicate oligomers and nanoparticles. To validate this, we performed solution-state ^29^Si NMR analysis of transparent sodium silicate solution having NaOH/Si = 0.54 and NaOH/Si = 0.60 (see Supplementary Fig. 14). NMR analysis for OH/Si = 0.60 detected three signals that can be assigned to Q^2^ ((SiO)_2_***Si***(O^−^)_2_), Q^3^ ((SiO)_3_***Si***(O^−^)), and Q^4^ ((SiO)_4_***Si***) Si species. The sharp signals for Q^2^ and Q^3^ are derived from small silicate species, while the broad peak for Q^4^ is indicative for formation of larger oligomers and/or nanoparticles. In addition to these three signals, the sodium silicate solution for OH/Si = 0.60 gave sharp signals for Q^0^ (***Si***(O^−^)_4_)) and Q^1^ ((SiO)***Si***(O^−^)_3_), indicating that larger silicate species decompose into monomer and dimer, respectively. Although actual synthesis temperatures and chemical compositions differ depending on synthesis conditions, Na/(Si + Al) ~0.57, appeared as a criterion in the decision tree (Fig. 3), is seemingly the boundary that decides what kind of soluble silicate species are dominant in liquid phase of a synthesis mixture. Collectively, the structure similarity in the synthesis clearly exists in the particular synthetic range, although it is not necessarily observed outside the applicable domain.

### Construction of a similarity network for zeolites

The machine learning models were solely trained for the synthesis descriptors, and the results can be used to rationalize physicochemical, structural, and empirical insights including solubility, Ostwald’s step rule, Löwenstein’s rule, and structural similarity (vide supra). From the viewpoint of the structural similarity, some building units, including *mor* and *d6r*, are likely more important than others. Indeed, not all of the building units should be equally significant, but some should correspond to critical motifs for the nucleation and growth of the crystals^35^. Because direct observation of these critical building units, if they exist, is technologically demanding, prioritization of the building units through fitting to the experimental results^35^ is the most natural approach. Thus, a numerical optimization algorithm was employed to transfer the similarities found in the multidimensional chemical space composed of the synthesis descriptors to the structural similarity of the crystals.

The synthesis similarity for a pair of zeolites can be quantified based on the center of the synthesizable domain for each zeolite (Fig. 1b and Supplementary Table 5). Variations in Si/Al_Reactant_ and Na/(Si + Al) were more influential than those of other synthesis descriptors upon calculating the distances between the synthesis conditions because the standardized synthesis descriptors were weighted by the importance in the XGBoost (Supplementary Fig. 4). The structural similarity of the zeolite structures was defined by one-dimensional vectors, often called fingerprints^52^, expressing the presence or absence of building units. Fingerprints can be used to predict the targeted features of chemical entities^52^ and automate retrosynthesis^53,54^. The most appropriate weighting (i.e., importance) of the building units that could excellently approximate the synthesis similarity was calculated by solving the optimization problem (described in the “Methods” section). As shown in Supplementary Fig. 15, the important building units with a high weight and small standard deviation were *sod*, *d8r*, *mor*, and *d6r*, which are consistent with the structural similarities observed in the decision tree (Fig. 3).

**Fig. 5:**
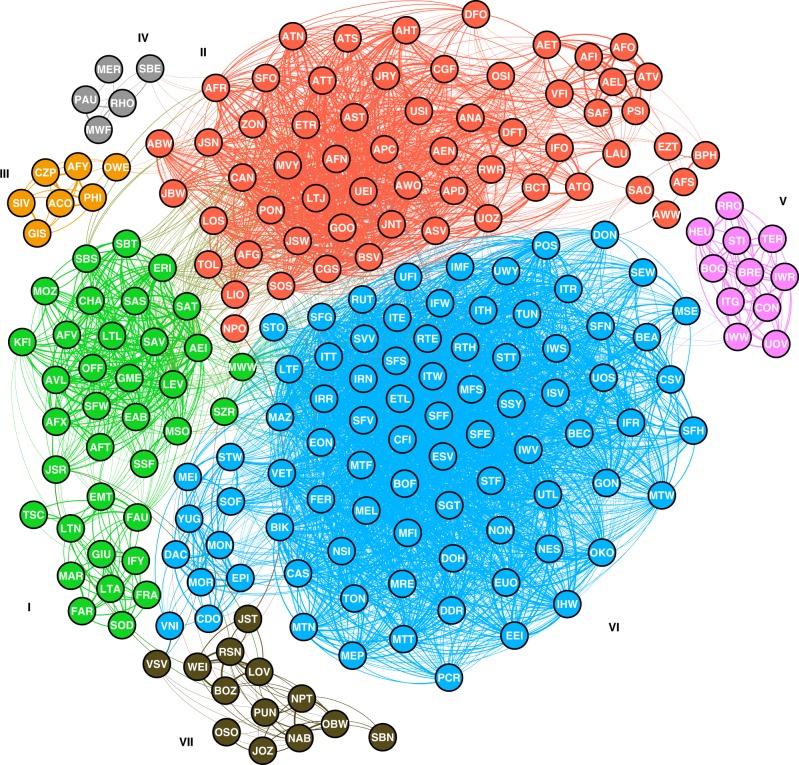
Similarity network for the zeolite structures. The layout of the network is decided by a force-directed algorithm. Communities are identified using a clustering algorithm based on the modularity optimization. To verify the weighting effects, another structural similarity network was constructed using identical weights for all building units (Supplementary Fig. 16)

To obtain additional insights, the structural similarities between all of the crystal structures of zeolites and zeotypes^55^ were calculated using the weighted fingerprint. The structural similarity is essentially the proximity in the multidimensional space composed of the structure descriptors (Fig. 1c)^56^. A similarity network of the zeolite structures was constructed by connecting structurally similar crystalline topologies as shown in Fig. 5, in which the layout of the nodes reflects the structural similarity^57^. To partition the network into sets of communities, a clustering algorithm was applied, which solely reflects the connections and their weights^58^. The clustering identified seven communities, which were colored and labeled as communities I–VII (see Fig. 5).

Most of the constituent structures of community I were relatively Al-rich (typically, Si/Al_Product_ < 3) zeolites. Lower part of community I was characterized by the common building unit *sod* scoring the highest importance (Supplementary Fig. 15). Some of the structures in the lower part of this sub-community (I_lower_) only occur naturally as minerals and have never been synthesized in the laboratory^55^. On the other hand, the most important building unit in the upper part of community I (I_upper_) was *d6r*, demonstrating its significance in the decision tree (vide supra). Many structures in this sub-community I_upper_ were categorized as the so-called ABC-6 stacking family. In this sub-community, **AEI**, **AFX**, **CHA**, **EAB**, **ERI**, **GME**, **LEV**, **OFF**, and **SFW** can be synthesized as aluminosilicate zeolites with OSDAs (Supplementary Table 6). In addition, several structures in sub-community I_upper_ can be formed in phosphate-based compositions, e.g., as aluminophosphate (AlPO_4_) zeotypes, including **AEI**, **AFT**, **AFX**, **AFV**, **AVL**, **CHA**, **ERI**, **LEV**, **LTL**, **SAS**, **SAT**, **SAV**, **SBS**, and **SBT**.

The phosphate-based zeotypes in community I were connected to structures in community II, which is dominated by other phosphate-based structures. In particular, a sub-community in community II consisting of **AEL**, **AET**, **AFI**, **AFO**, **ATV**, **PSI**, **SAF**, and **VFI** possessed high structural similarity arising from the common *afi* and *bog* units (see Supplementary Fig. 1). Similar to *d6r*, the structures of *afi* and *bog* units built from *4r* and *6r* may have structural compatibility with aluminophosphates. The constituent structures of community III were also phosphate-based structures but did not contain *6r* except for **OWE**. Community IV reflected the importance of the *d8r*-containing **RHO** and **PAU** structures, which are considered as members of the so-called **RHO**-family^32^. The structural similarity of the **RHO**-family provided a guideline for the successful synthesis of new zeolites in this family, including PST-20 and PST-25^32^, remarkably demonstrating the synthesis–structure relationship. Inclusion of computationally generated hypothetical structures into the similarity network can give further insights for their synthesis and may lead to the discovery of new zeolites, although this is beyond the scope of the current study.

The major building units in community V were *bre* and *sti*. One of the interesting features of this community was that it contains naturally occurring aluminosilicate zeolites, including **BOG**, **BRE**, **HEU**, **TER**, and **STI**. More importantly, all of the structures in this community had topologically multidimensional channels in two or more directions^59^, even though rings larger than *6r* were not considered as the structure descriptors in this study. Furthermore, all of the structures except **TER** and **BRE** have interconnected channels with different pore apertures (see Supplementary Table 7). The fact that community V compiled such structures suggests that *bre* and *sti* are likely related to the formation of multipore zeolites.

Community VI was dominated by high-silica zeolites and zeotypes containing *5r*. Insights can be acquired from the locations of the nodes in Fig. 5. For example, **CAS**–**NSI** and **STF**–**SFF** are closely related structures constructed with different stacking sequences of layer-like building units^60,61^. The structures clustered at the bottom of community VI (**DDR**, **DOH**, **MEL**, **MEP**, **MFI**, **MRE**, **MTF**, **MTN**, **MTF**, **MTT**, **NON**, **SGT**, and **TON**) are all obtained as pure-silica zeolites from Si source, water, and OSDAs with hydroxides, demonstrating their synthesis similarity. Community VII was composed of the so-called unfeasible structures possessing *3r*, *lov*, and/or *vsv* units that have proven to be too strained for silicate structures^62^. The crystallization of such highly strained structures requires atypical tetrahedral atoms, such as Be, Zn, and Ge to relax the structural distortion.

### Application of the similarity network to zeolite synthesis

**Fig. 6:**
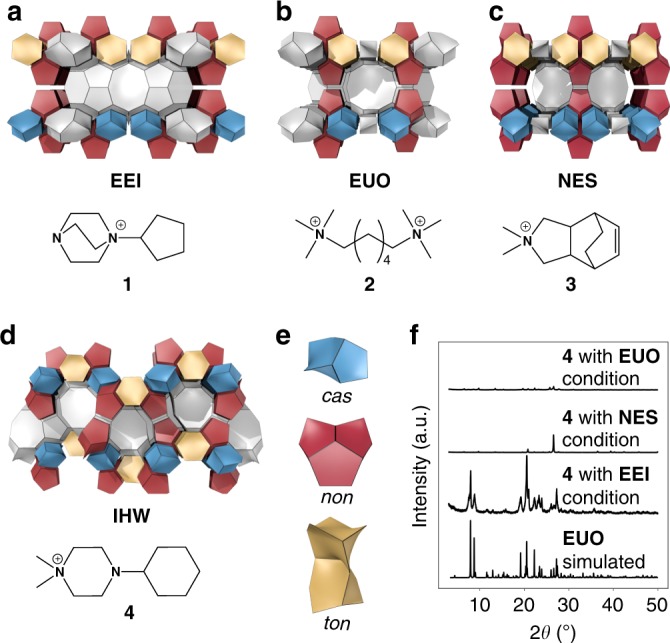
Crossover synthesis experiments for **EEI**, **EUO**, **NES**, and **IHW**. **a**–**d** Crystal structures of **EEI** (**a**), **EUO** (**b**), **NES** (**c**), and **IHW** (**d**) with typical OSDAs (**1**–**4**) used for their syntheses. **e** Building units (*cas*, *non*, and *ton*) found in the four structures. **f** Powder XRD patterns of the products synthesized using **4** as an OSDA under the typical synthesis conditions for **EEI**, **EUO**, and **NES** (see Supplementary Table 8). Thermogravimetric analysis confirmed that a single OSDA was occluded in the cage of the **EUO** prepared from **4** under **EEI** conditions

To provide further evidence for the applicability of the similarity network, crossover experiments of zeolite syntheses using OSDAs were performed. Among structurally related zeolites, the **EEI**–**EUO**–**NES** zeolite family as well as **IHW** were selected (Fig. 6), as they were located in community VI (Fig. 5) with close proximity. The structural similarity between **EEI**, **EUO**, and **NES** has been previously recognized owing to their similar layered motifs and common building units^63^. Nevertheless, **IHW**^64^ has not been considered as a member of this zeolite family, and its synthesis conditions are notably different from the other structures (see Supplementary Table 8). The biggest difference in the synthesis of **IHW** compared to the other structures is the use of fluoride media, which leads to substantially different chemistry compared to its hydroxide counterpart. The crossover experiments were carried out by mimicking the typical synthesis conditions for **EEI**^65^, **EUO**^66^, and **NES**^67^, but replacing the OSDAs originally used (**1**–**3**) with **4**, which was reported to crystallize **IHW**^64^ (see Fig. 6).

Although the explored three conditions have notably similar Si/(Si + Al), OSDA/(Si + Al), and H_2_O/(Si + Al) ratios, the other parameters including type of inorganic cations, heating conditions, and used chemicals are different, resulting in different products (Table 1). The employment of **4** with the synthesis conditions for **NES** yielded a brown suspension, implying that Hoffman degradation of **4** occurred during hydrothermal treatment at 180 °C for 406 h. The **NES** synthesis conditions seemed to be too harsh for **4** and hindered the involvement of **4** in the crystallization of zeolites. The relatively long heating time seemingly led to the formation of α-quartz, as indicated by the powder XRD pattern in Fig. 6f. The synthesis conditions for **EUO** in the presence of **4** resulted in the formation of a brown suspension, again suggesting the degradation of **4**. The XRD pattern of the solid product confirmed the presence of a trace amount of **MOR**. Indeed, the decision tree in Fig. 3 predicts the formation of **MOR** under these conditions. On the other hand, the lower temperature in the typical synthesis conditions for **EEI** was apparently appropriate for **4**, judging from the resulting white product that was identified as **EUO** (see the XRD pattern in Fig. 6f). The fact that the same OSDA can direct the formation of structurally similar **IHW** and **EUO** zeolites by mimicking the synthesis conditions for **EEI** confirms the synthesis similarity of the structures and the applicability of the synthesis–structure relationship beyond the OSDA-free synthesis of zeolites.

**Table 1:** Explored synthesis conditions for crossover experiments

Original product	OSDA 4^a^	Si^a^	Na^a^	K^a^	F^a^	H_2_O^a^	Temperature (°C)	Heating time (h)
**EEI** ^65^	0.20	0.996	–	0.05	–	41	160	336
**EUO** ^66^	0.22	0.957	0.33	–	–	50	200	22.5
**NES** ^67^	0.24	0.952	0.27	–	–	47	180	406

^a^Chemical composition divided by (Si + Al)

## Discussion

Previous studies have struggled to provide a clear description of the synthesis–structure relationship in materials, such as zeolites that are formed through kinetically controlled pathways. This study takes advantage of machine learning techniques to recognize patterns hidden in the experimental data. The knowledge extracted from the machine learning models rationalizes physicochemical, structural, and empirical insights into the zeolite chemistry. Proper synthesis descriptors are identified from the training with quantitative importance, which is subsequently transferred to recognize the primary structure descriptors. Based on the synthesis and structure descriptors with rationalized importance, a similarity network can be constructed by including the zeolite structures outside of the dataset used for machine learning, demonstrating the broad applicability of the approach. The similarity map revealed previously overlooked structural similarities, which were verified with crossover experiments. The current approach can be applied to any materials, including those formed through kinetically controlled pathways. The guided synthesis of materials based on the synthesis–structure relationship can be used to not only rationalize the known syntheses and discover novel materials, but also to increase the size and diversity of the available datasets, which are remarkably important for improving the linkages between synthesis descriptors and structure descriptors.

## Methods

### Dataset

Although several zeolites have been synthesized in the presence of seed crystals, OSDAs, and fluoride, the present study collected experimental data only from OSDA-free syntheses of aluminosilicate zeolites in hydroxide media without seeds. Records of syntheses that resulted in multiple crystalline phases under the same conditions were excluded, with a few exceptions. Synthesis of **EMT** zeolite under OSDA-free conditions often yields **FAU** zeolite as an impurity. Considering the limited reports of OSDA-free synthesis of pure **EMT**^68^ in the dataset, both **EMT** and **EMT**–**FAU** intergrowths were regarded as **EMT**–**FAU**. For similar reasons, the records for synthesis of **TON** and mixtures of **TON** and cristobalite were regarded as **TON**. Syntheses of **ERI** and **OFF** were expressed as **ERI**–**OFF** because they are typically formed as intergrown crystals in OSDA-free synthesis. **ABW**, **EON**, **GME**, **LTN**, and **MAZ** were omitted from the dataset because there are only few synthetic reports of pure phase formation. Literature used as the data source is summarized in Supplementary Table 1. It largely relies on a review by Oleksiak and Rimer^22^ that exhaustively summarized reliable literatures. We also added several uncovered experiments, which were tested by machine learning techniques used in this study for consistency with the review.

### Machine learning

The dataset was divided into a training set (80%) and a test set (20%) to tune and validate the machine learning models. Supervised machine learning models including decision tree, random forest, and support vector machine models were constructed using scikit-learn^69^. Five-fold cross-validation was used to train the machine learning models and to optimize their hyperparameters with a grid search of the candidate values presented in Supplementary Tables 9–11. The models based on XGBoost were constructed using its Python interface^70^. The hyperparameters of XGBoost were tuned with Bayesian optimization using Gaussian Processes^71^ for the candidate values listed in Supplementary Table 12. Continuous features were standardized upon training and prediction of the machine learning models.

### Metropolis Monte Carlo simulation

The Metropolis Monte Carlo method at a finite temperature^43^ was employed to estimate the Gibbs free energies of zeolites. Zeolite models with specified Si/Al_Product_ having Na^+^ were first created by randomly placing Al and counter cations while avoiding the formation of Al–O–Al^42,48^ from idealized crystal models^55^. Then, the structures were optimized using an interatomic potential tuned for zeolites^72^ with GULP software^73^. After optimization for 10 steps, the randomly chosen AlO_4_ and its corresponding Na^+^ cation were swapped with another randomly selected TO_4_(Na^+^). If the energy decreased following the structure optimization for 10 steps, the swap was accepted. Otherwise, the swap was accepted with the following probability:

1 $$P = \exp \left( { - \frac{{\Delta U}}{{k_{\mathrm{B}}T}}} \right)$$

where −Δ*U* is the difference in energy before and after swapping, and *k*_B_ is the Boltzmann constant. The temperature (*T*) was 300 K. This cycle of swapping and structure optimization was repeated 1000 times. The Gibbs free energy of a zeolite with a given composition was estimated by applying the following equation:

2 $$G = - k_{\mathrm{B}}T\,\ln \left[ {\mathop {\sum}\limits_i {\exp \left( { - \frac{{E_i}}{{k_{\mathrm{B}}T}}} \right)} } \right]$$

where *E*_*i*_ is the energy of the *i*th atomic configuration. Mean and standard deviation of *G* were calculated from five independent simulations.

### Analyses of synthesis and structural similarities

Sequential least-squares programming^74^ was used to solve the following optimization problem:

3 $${\mathrm{minimize}}_{{\mathbf{w}}_i}\mathop {\sum }\limits_i \mathop {\sum }\limits_{i \ne j} \left[ {\left( {{\mathbf{xr}}_i - {\mathbf{xr}}_j} \right)^2 - \left( {{\mathbf{w}}_i{\mathbf{u}}_i - {\mathbf{w}}_j{\mathbf{u}}_j} \right)^2} \right]$$

where *i* iterates over all of the crystal structures of interest, **x** is the importance of the synthesis descriptor computed by XGBoost, **r**_*i*_ is a representative value of the synthesis descriptors in structure *i*, **u**_*i*_ is the binary vector expressing the presence or absence of the building units in structure *i*, and **w**_*i*_ is the weight of the building units. The central synthesis condition, **r**_*i*_, is the geometric median of the synthetic reports for each zeolite structure in the standardized chemical space weighted by its importance in XGBoost.

Crystal structures of zeolites were retrieved from the database^55^ excluding those with defects. A complete list of the building units is presented in Supplementary Fig. 1. Rings larger than a six-membered ring (*6r*) were excluded because their large degree of freedom allows for diverse bond angles and distortions in the crystal structures. Subgraph isomorphism was performed using the VF2 algorithm^75^ to detect building units in the crystal structures. The unit cells were expanded to 2 × 2 × 2 super cells. For the topological analysis, tetrahedral atoms were regarded as nodes and bridging oxygen atoms were regarded as links. Structural similarities between crystal topologies were calculated with the Tanimoto similarity index^56^ using the presence (or absence) of building units as the fingerprint. The fingerprint was weighted by the corresponding importance, ***w***_*i*_. Unknown weights of building units were filled with the average of the known weights. The similarity network was constructed by linking a pair of crystals with a Tanimoto similarity of more than 0.7. The largest connected network was partitioned by modularity optimization^58^ and visualized using the ForceAtlas2 algorithm^57^.

### Chemical synthesis

See details in Supplementary Methods.

## Supplementary information

Supplementary Information

## Data Availability

The data that support the findings of this study are available within the Article and its Supplementary Information, or from the corresponding authors on reasonable request.
