# Supplementary material for: Linking synthesis and structure descriptors from a large collection of synthetic records of zeolite materials
Source: Nat Commun. 2019 Oct 1;10:4459. doi: 10.1038/s41467-019-12394-0 (PMC6773695; doi:10.1038/s41467-019-12394-0)
Supplement: Supplementary file 1 — Supplementary Information [file 41467_2019_12394_MOESM1_ESM.pdf]

Supplementary Information

Linking synthesis and structure descriptors from a large collection of synthetic records of zeolite materials

Muraoka et al.

Supplementary Methods

Chemicals. The chemicals used for the synthesis of organic compounds and zeolites were purchased from commercial suppliers and used as received. 1-Cyclohexylpiperazine was obtained from Tokyo Chemical Industry Co., Ltd. Iodomethane, potassium carbonate, acetone, diethyl ether, chloroform, DOWEX MONOSPHERE 550A (OH) anion exchange resin, hydrochloric acid (0.01 M), deuterium oxide, sodium hydroxide, and potassium hydroxide were obtained from Wako Pure Chemical Industries, Ltd. Ludox HS-40 colloidal silica, aluminum hydroxide and Ludox HS-30 colloidal silica were purchased from Sigma-Aldrich Co., LLC.

Solution-state ^{29}Si NMR analysis. Sodium hydroxide was dissolved in a mixture of deionized water and deuterium oxide, followed by the dropwise addition of Ludox HS-40 colloidal silica. The final composition of the mixture was 1.0 SiO_2: x NaOH: 20 H_2O: 8 D_2O. The mixture was agitated at ambient temperature for 1 d. The resulting colorless transparent solution was casted to solution-state ^{29}Si NMR analysis with a relaxation time of 30 s.

Synthesis of 4. A mixture of 1-cyclohexylpiperazine (25.0 g, 149 mmol), potassium carbonate (20.5 g, 149 mmol), and acetone (300 mL) was cooled to 0 °C in an ice bath. Iodomethane was slowly added dropwise to the cooled mixture over 20 min while mixing. After adding an additional amount of acetone (200 mL), the resulting mixture was protected from light and stirred for 24 h at ambient temperature. Potassium salts were removed by filtration. The solvent in the obtained filtrate was evaporated to yield the crude product. The product was washed with diethyl ether and then extracted with chloroform. The 4-cyclohexyl-1,1-dimethylpiperazin-1-ium (**4**) iodide obtained after removal of organic solvent by rotary evaporation was dried at ambient temperature. ^{1}H NMR (400 MHz, $CDCl_3$, TMS): δ (ppm) 3.69–3.66 (m, 4H), 3.57 (s, 6H), 2.90 (t, J = 5.0 Hz, 4H), 2.38 (t, J = 6.8 Hz, 1H), 1.83–1.80 (m, 4H), 1.66–1.06 (m, 6H). ^{13}C NMR (101 MHz, $CDCl_3$, TMS): δ (ppm) 62.8, 52.1, 42.8, 28.9, 25.9, 25.4. The organic salt in iodide form was converted to the hydroxide form using a DOWEX

MONOSPHERE 550A (OH) anion exchange resin. The aqueous solution was concentrated and titrated with a 0.01 M HCl aqueous solution to quantify the concentration of **4** in hydroxide form.

Synthesis of zeolites. A mixture of alkali metal cation (either NaOH or KOH), aqueous solution of **4** (in hydroxide form), and water was used to dissolve aluminum hydroxide. After adding colloidal silica to the dissolved aluminate solution, the mixture was stirred for 30 min at ambient temperature. The mixture was then transferred to an autoclave. After heating at targeted temperature for desired time, the autoclave was cooled to ambient temperature with tap water. The solid product was recovered by filtration and washed with deionized water until pH of the filtrate decreased to 8. The solid product was dried at 80 °C overnight.

Characterization. Solution-state nuclear magnetic resonance (NMR) was recorded on a JEOL JNM-ECZ400S. Powder X-ray diffraction (XRD) patterns were obtained using a Rigaku Ultra IV with CuKα radiation (40 kV, 40 mA). Thermogravimetric analysis (TGA) was performed on a PU 4K (Rigaku) with a heating rate of 10 K min^{-1} using a mixture of 10% O_2 and 90% He as the carrier gas.

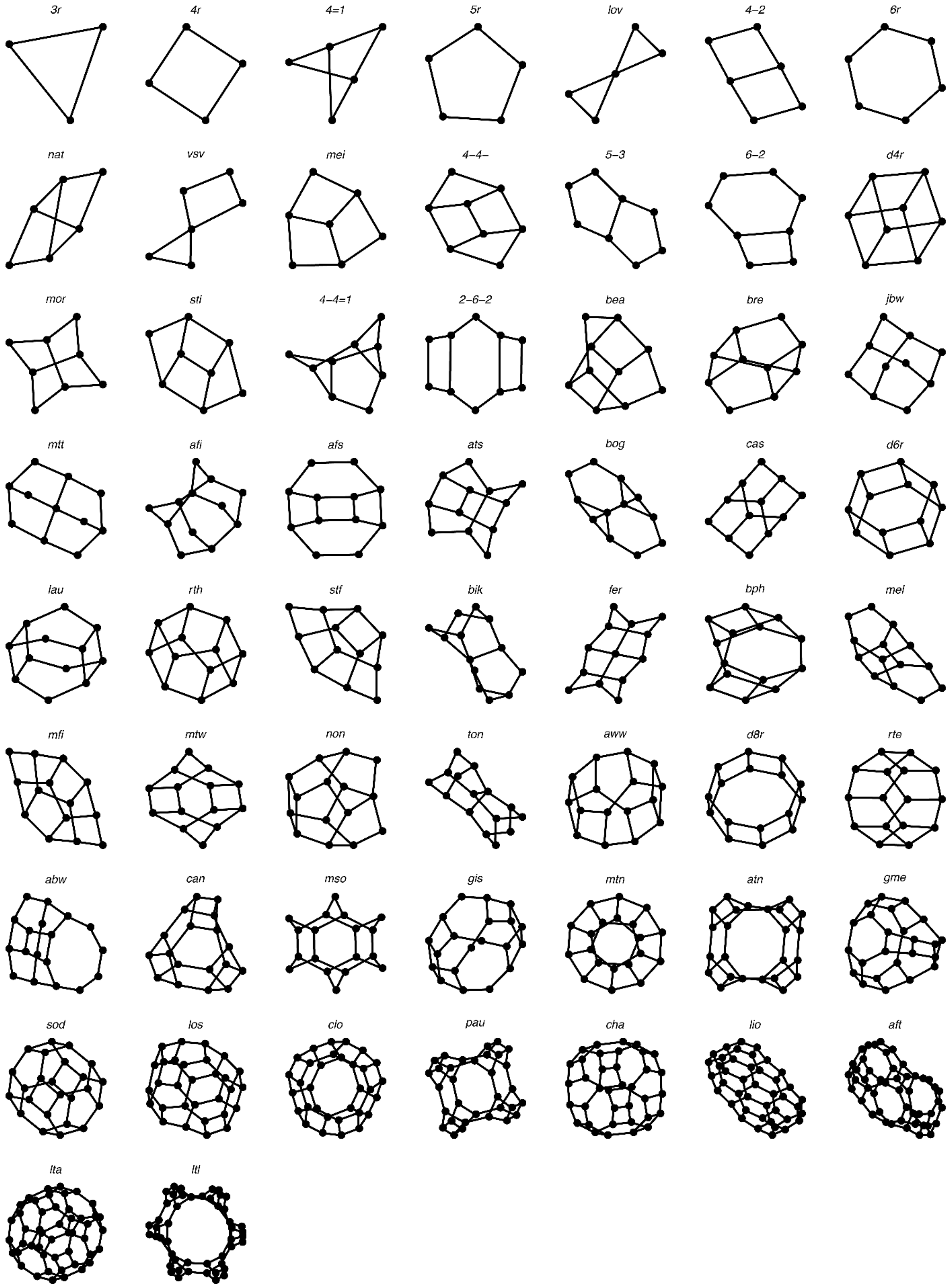

Supplementary Fig. 1 | Topologies of building units considered in this study. Nodes correspond to tetrahedral atoms, while links correspond to oxygen atoms.

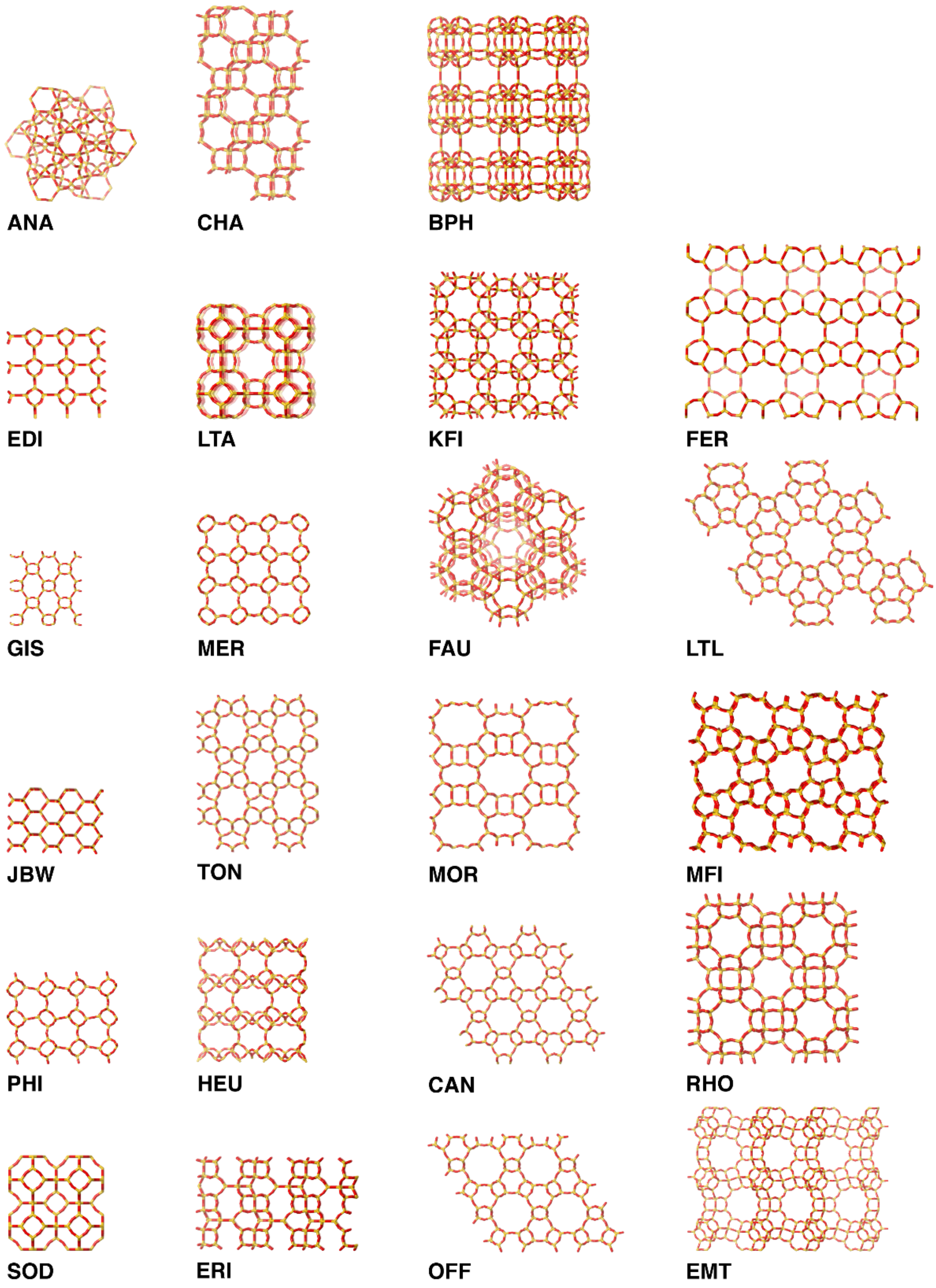

Supplementary Fig. 2 | Crystals structures of zeolites in the present dataset. Zeolites having different crystalline phases can be synthesized by only slight changing the synthesis descriptors such as chemical compositions of raw materials, heating time, heating temperature, and types of organic molecules called organic structure-directing agents (OSDAs).

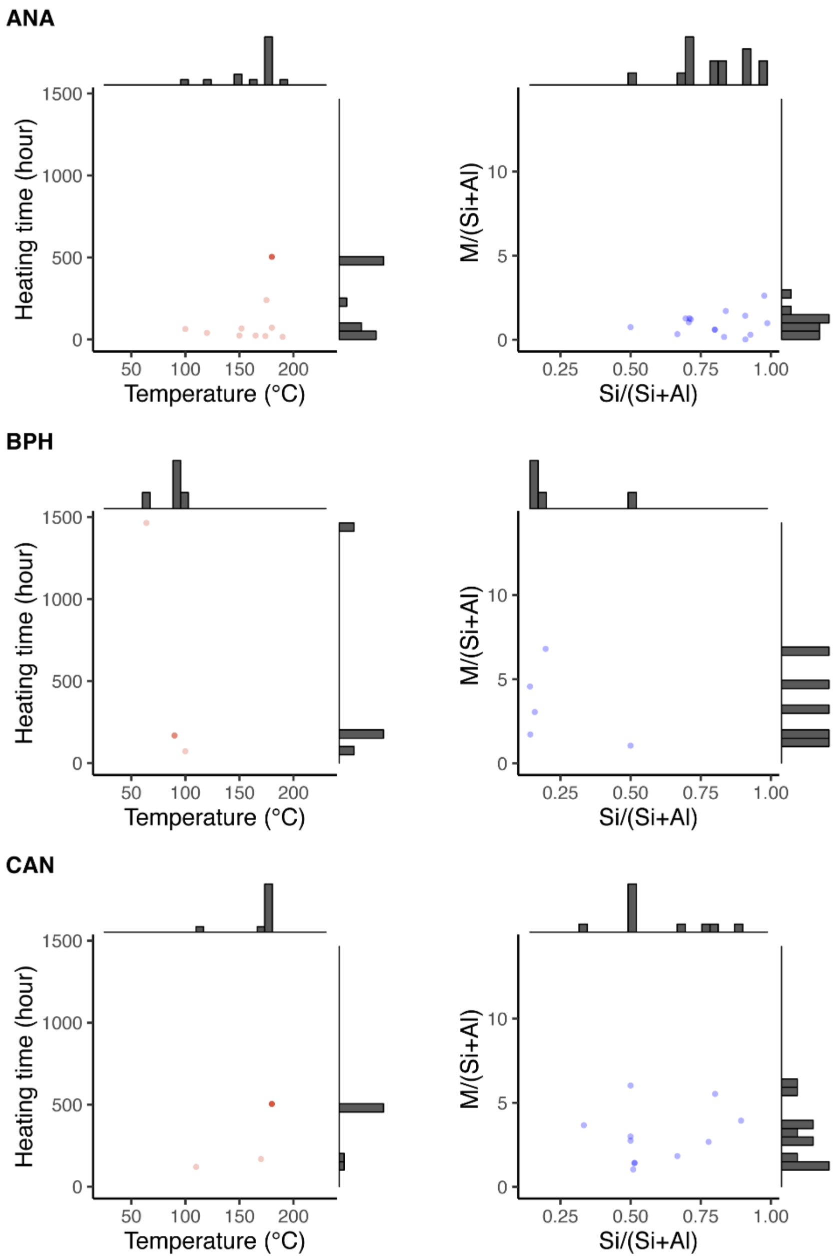

Supplementary Fig. 3 | Distribution of the dataset for each phase. Heating time versus temperature (left) and M/(Si+Al) versus Si/(Si+Al) (right).

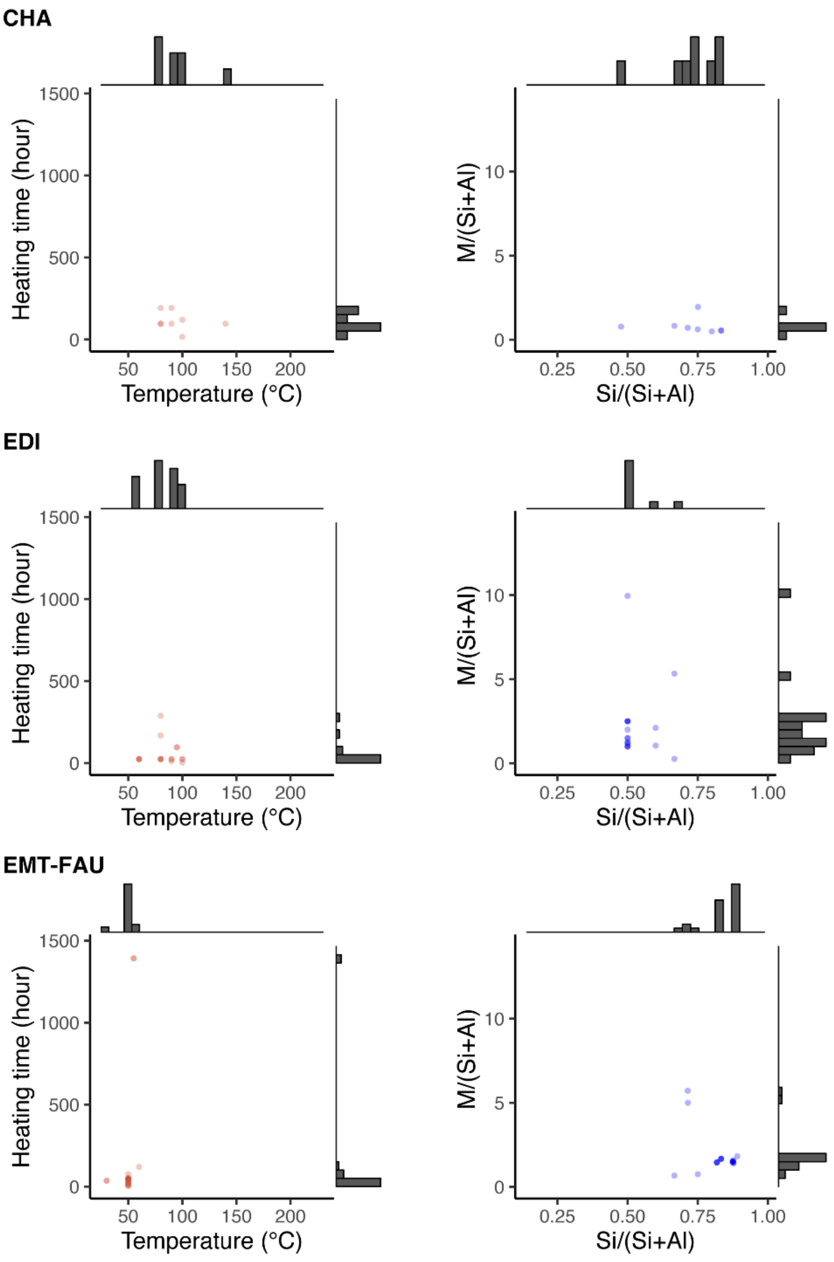

Supplementary Fig. 3 (continued) | Distribution of the dataset for each phase. Heating time versus temperature (left) and M/(Si+Al) versus Si/(Si+Al) (right).

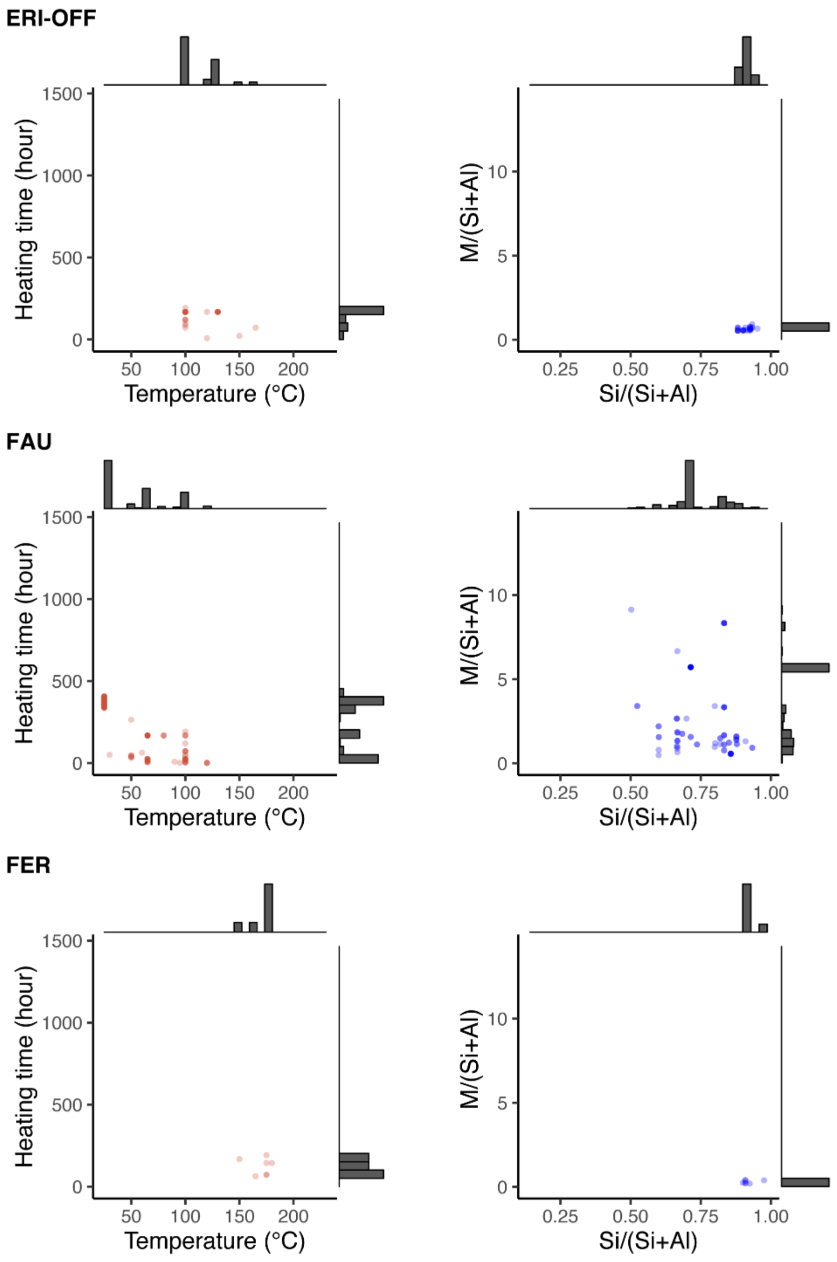

Supplementary Fig. 3 (continued) | Distribution of the dataset for each phase. Heating time versus temperature (left) and M/(Si+Al) versus Si/(Si+Al) (right).

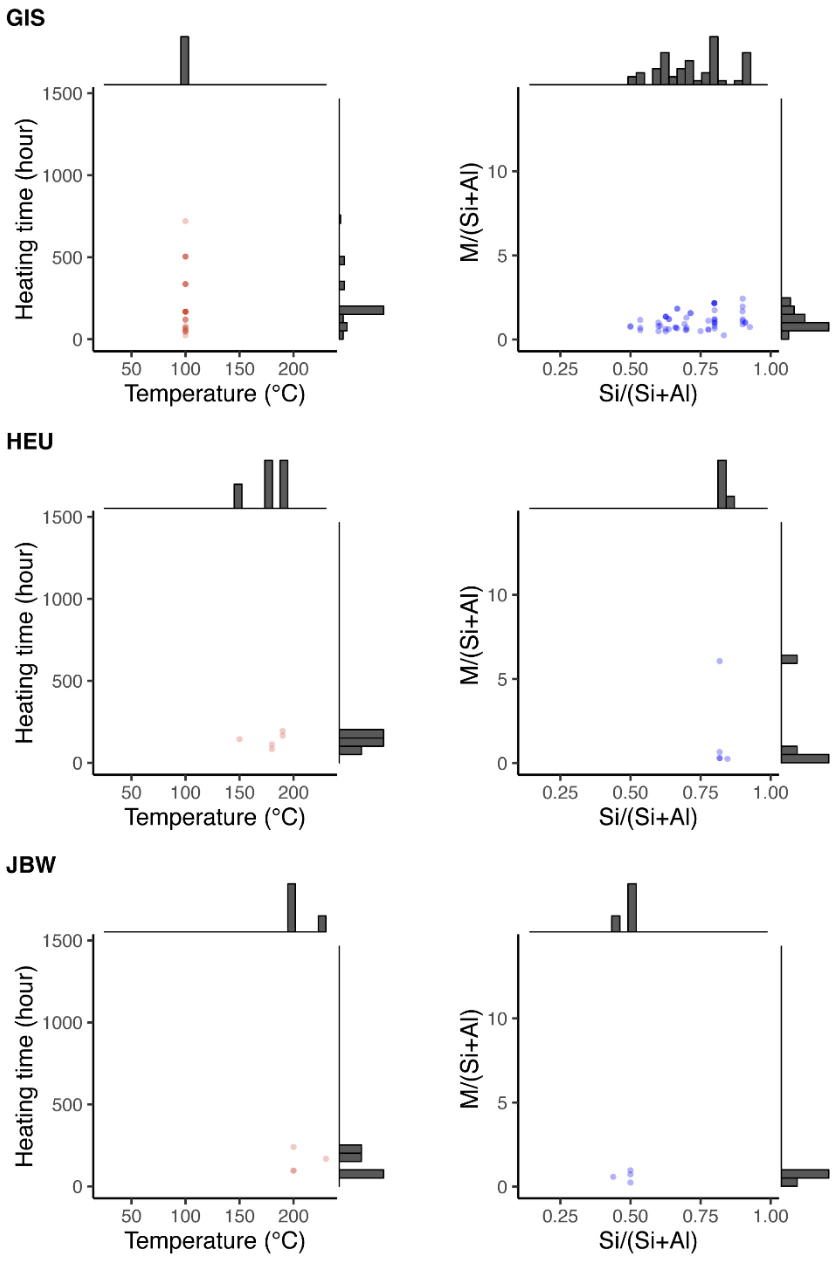

Supplementary Fig. 3 (continued) | Distribution of the dataset for each phase. Heating time versus temperature (left) and M/(Si+Al) versus Si/(Si+Al) (right).

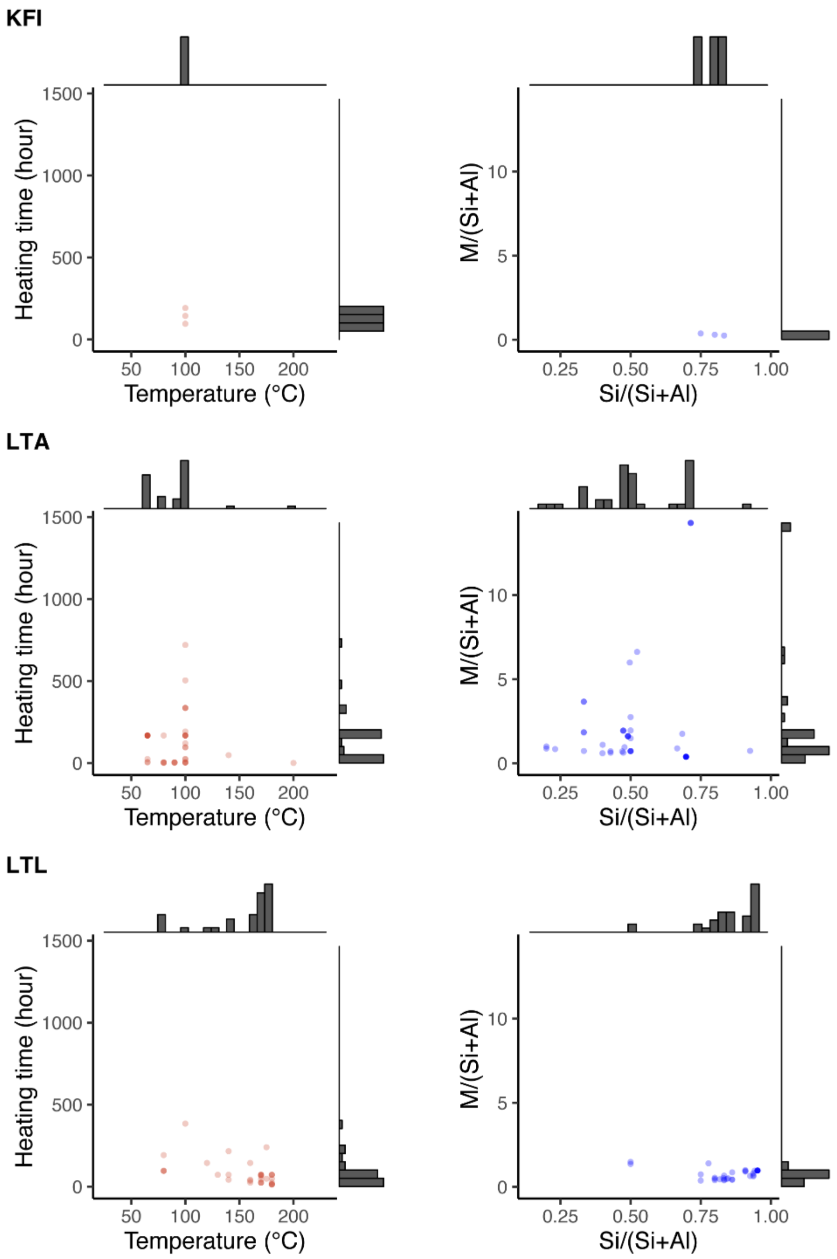

Supplementary Fig. 3 (continued) | Distribution of the dataset for each phase. Heating time versus temperature (left) and M/(Si+Al) versus Si/(Si+Al) (right).

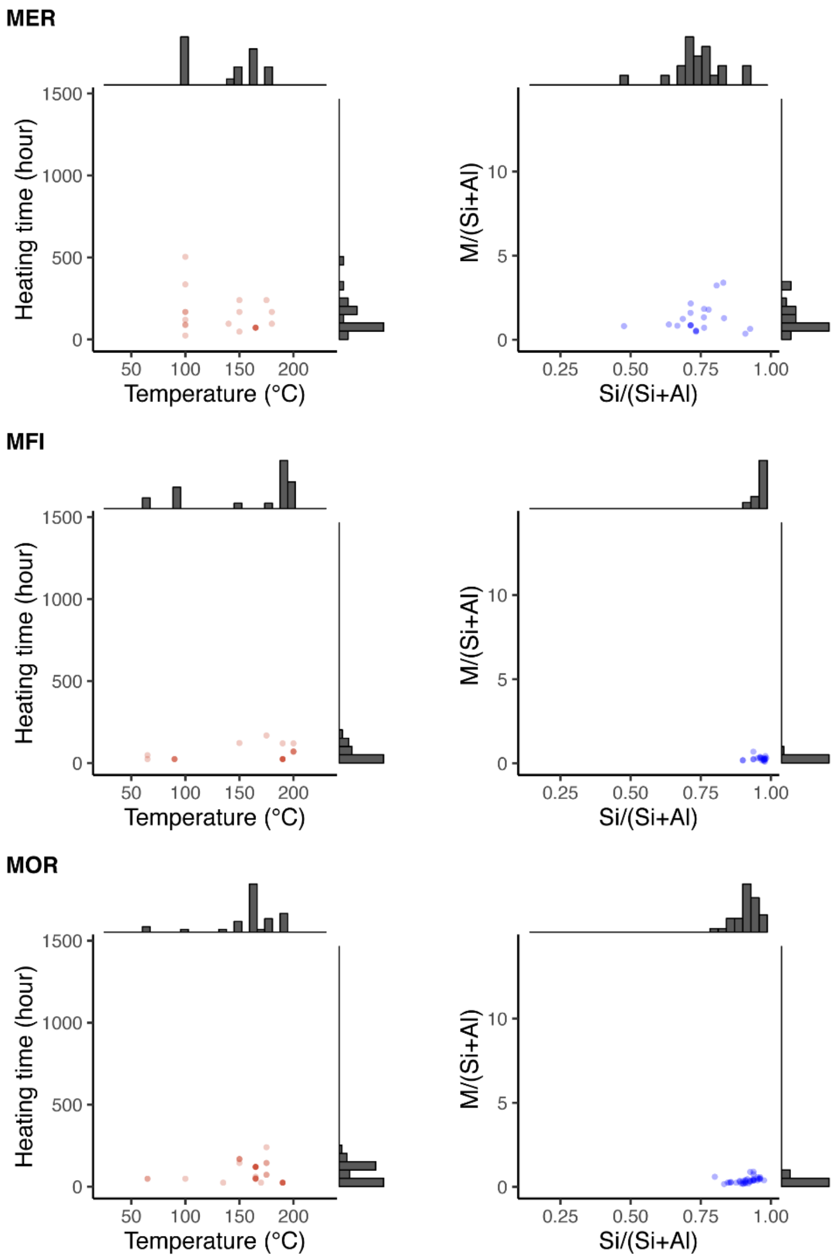

Supplementary Fig. 3 (continued) | Distribution of the dataset for each phase. Heating time versus temperature (left) and M/(Si+Al) versus Si/(Si+Al) (right).

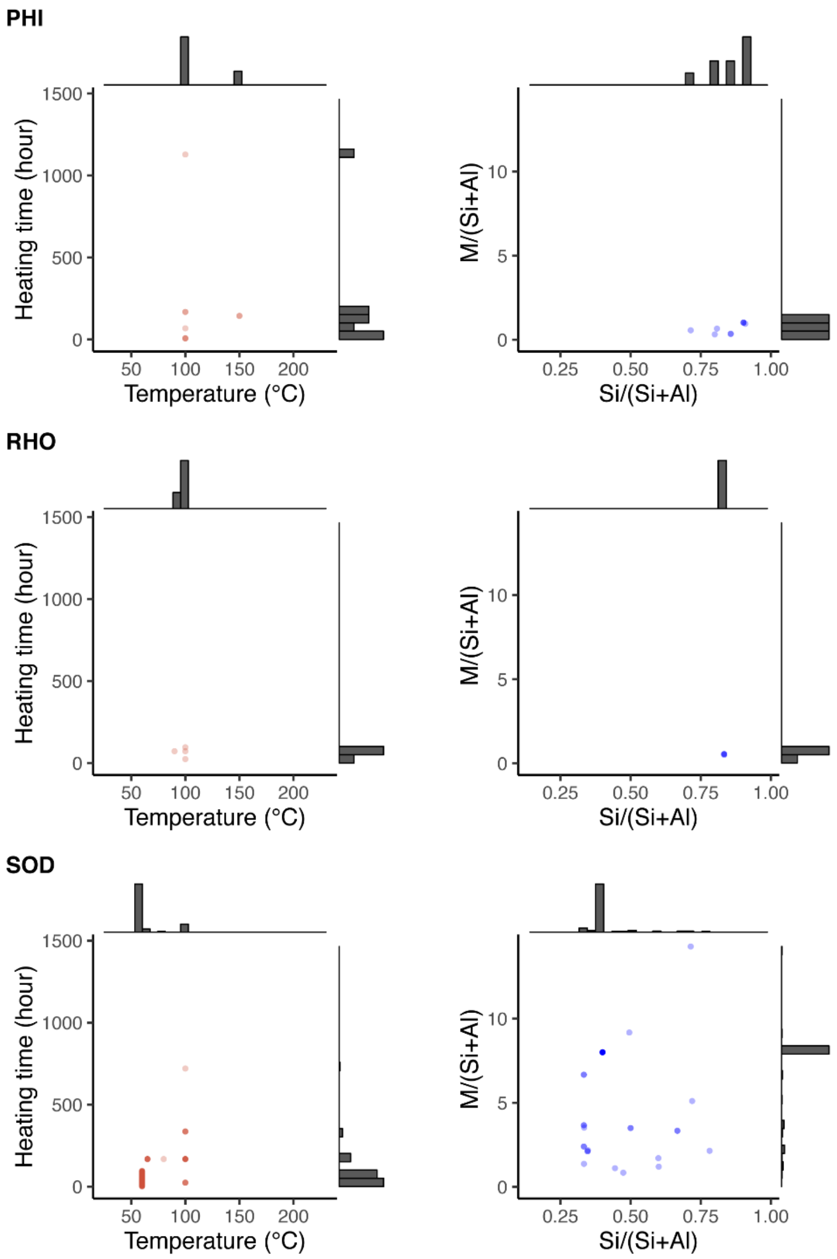

Supplementary Fig. 3 (continued) | Distribution of the dataset for each phase. Heating time versus temperature (left) and M/(Si+Al) versus Si/(Si+Al) (right).

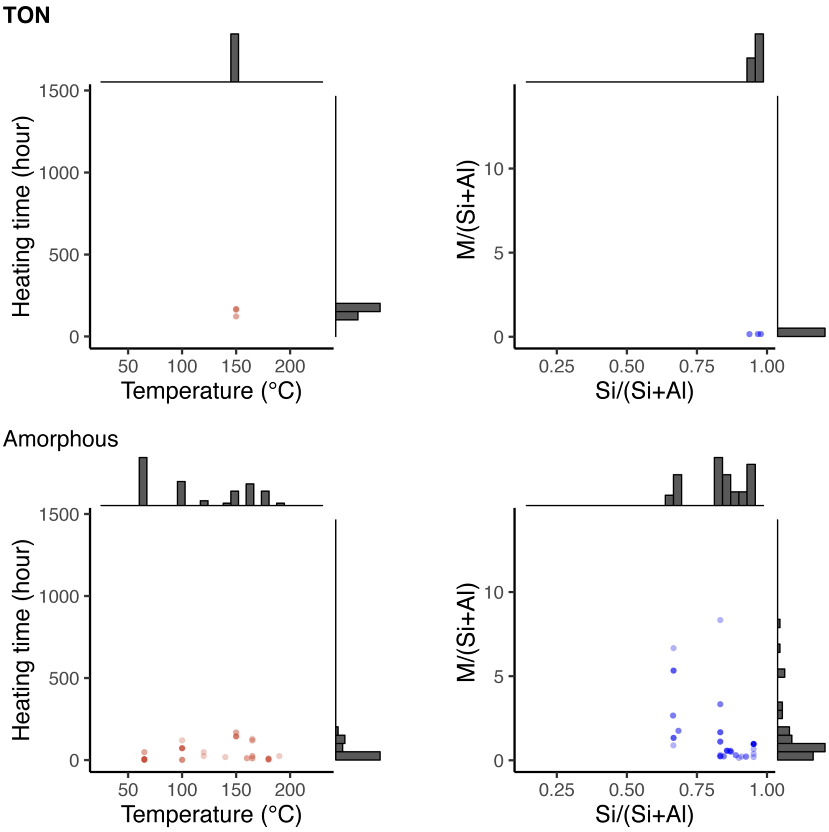

Supplementary Fig. 3 (continued) | Distribution of the dataset for each phase. Heating time versus temperature (left) and M/(Si+Al) versus Si/(Si+Al) (right).

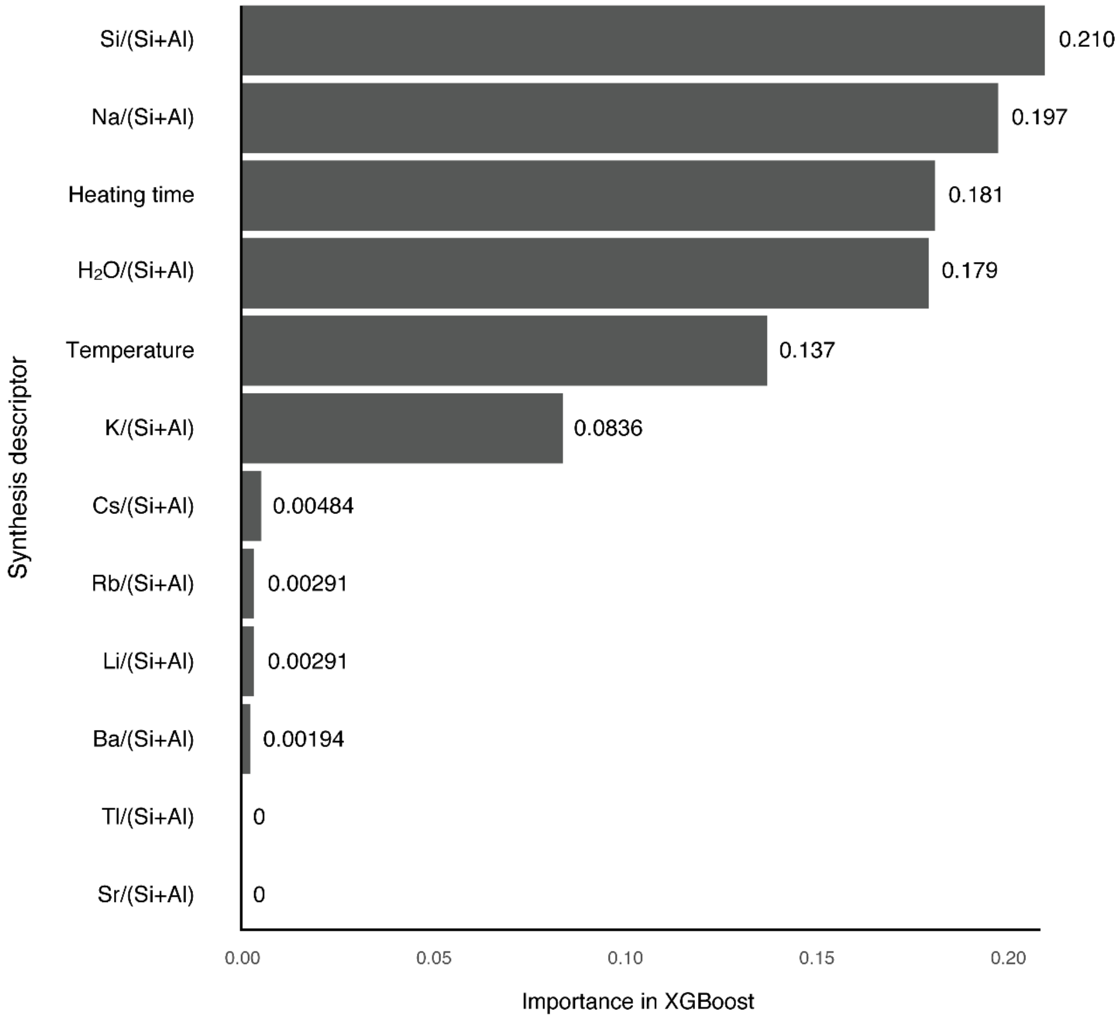

Supplementary Fig. 4 | Importance of the synthesis descriptors derived from XGBoost.

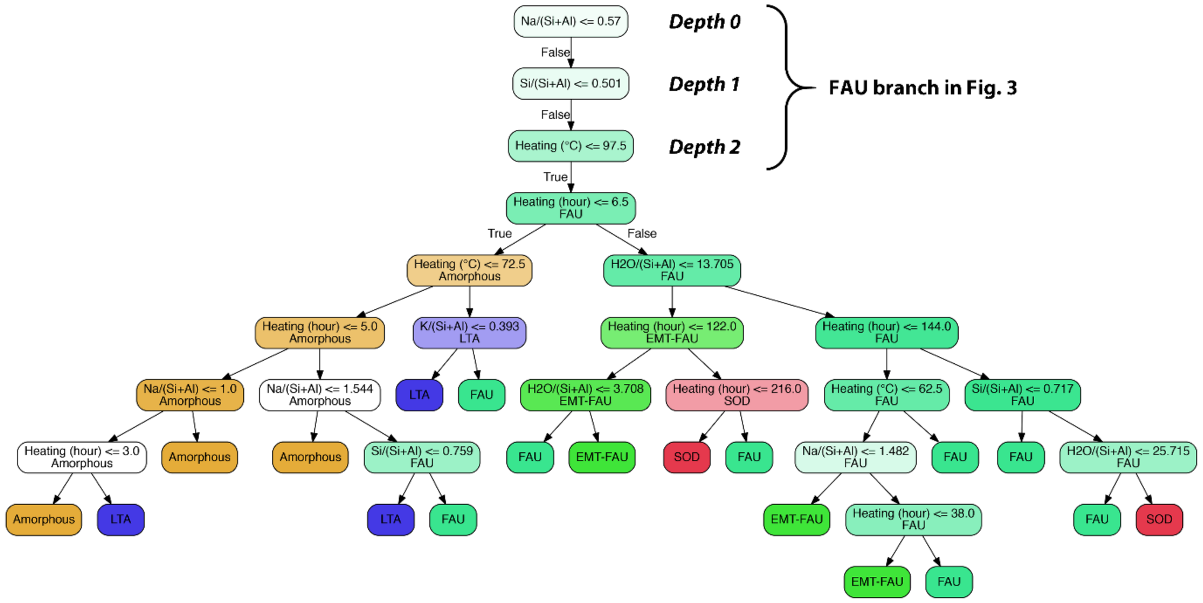

Supplementary Fig. 5 | The complete decision tree beneath the FAU branch shown in Fig. 3 in the main article. Below the depth of 3, arrows point left leaves satisfy a decision criterion shown in its root, while those point right do not.

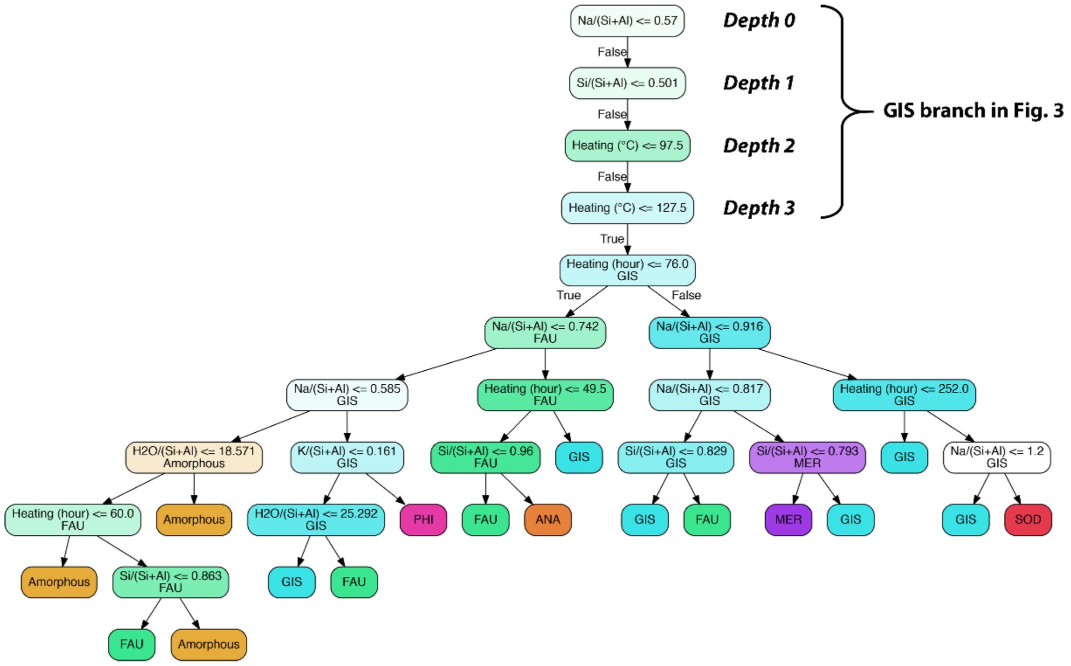

Supplementary Fig. 6 | The complete decision tree beneath the GIS branch shown in Fig. 3 in the main article. Below the depth of 4, arrows point left leaves satisfy a decision criterion shown in its root, while those point right do not.

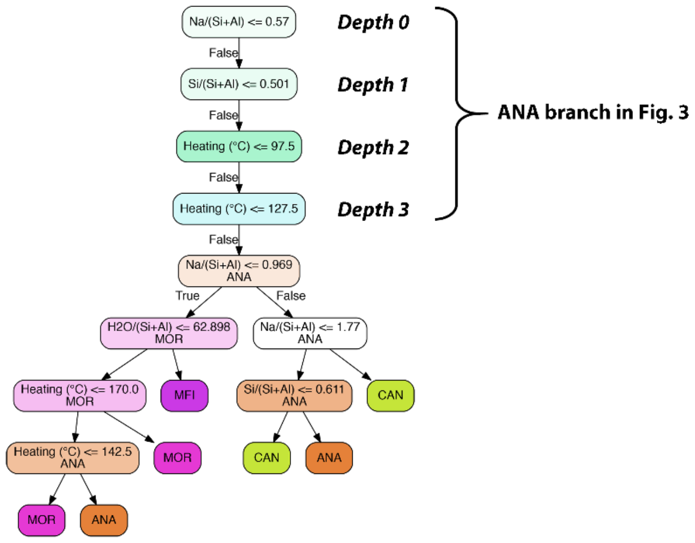

Supplementary Fig. 7 | The complete decision tree beneath the ANA branch shown in Fig. 3 in the main article. Below the depth of 4, arrows point left leaves satisfy a decision criterion shown in its root, while those point right do not.

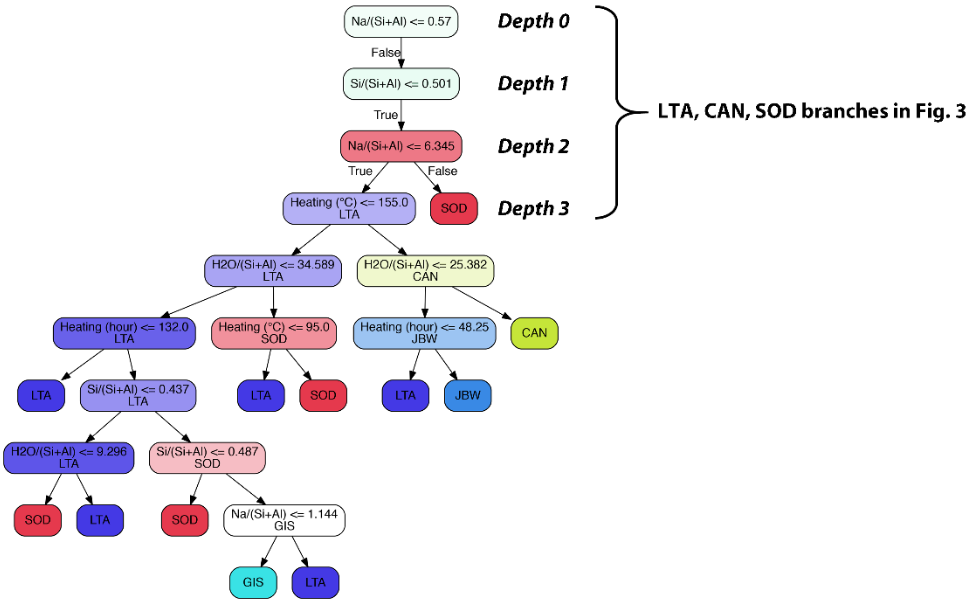

Supplementary Fig. 8 | The complete decision tree beneath the LTA and CAN branches shown in Fig. 3 in the main article. Below the depth of 4, arrows point left leaves satisfy a decision criterion shown in its root, while those point right do not.

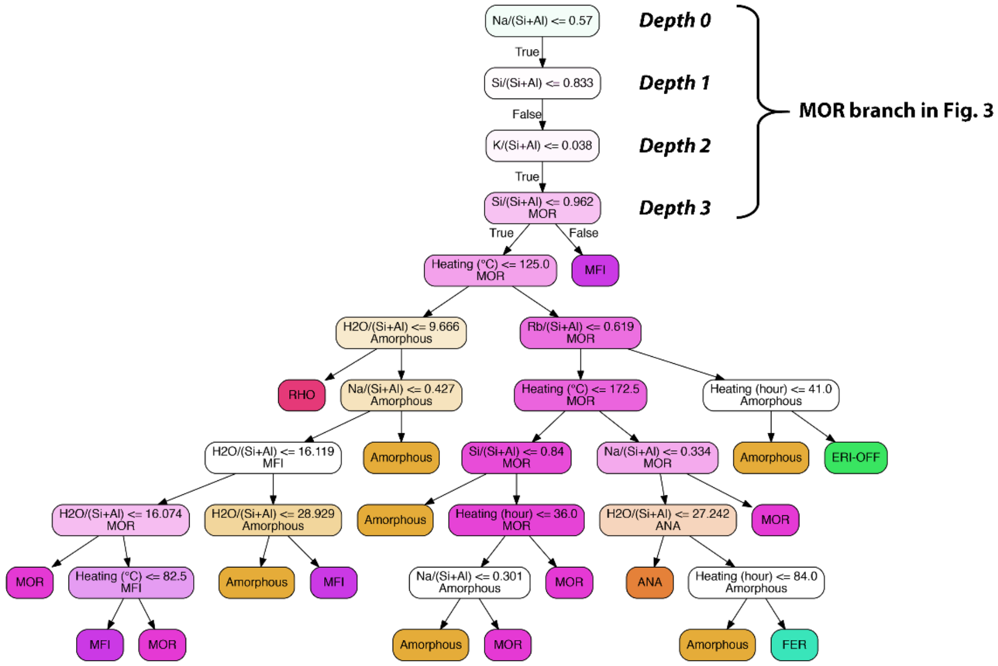

Supplementary Fig. 9 | The complete decision tree beneath the MOR branch shown in Fig. 3 in the main article. Below the depth of 4, arrows point left leaves satisfy a decision criterion shown in its root, while those point right do not.

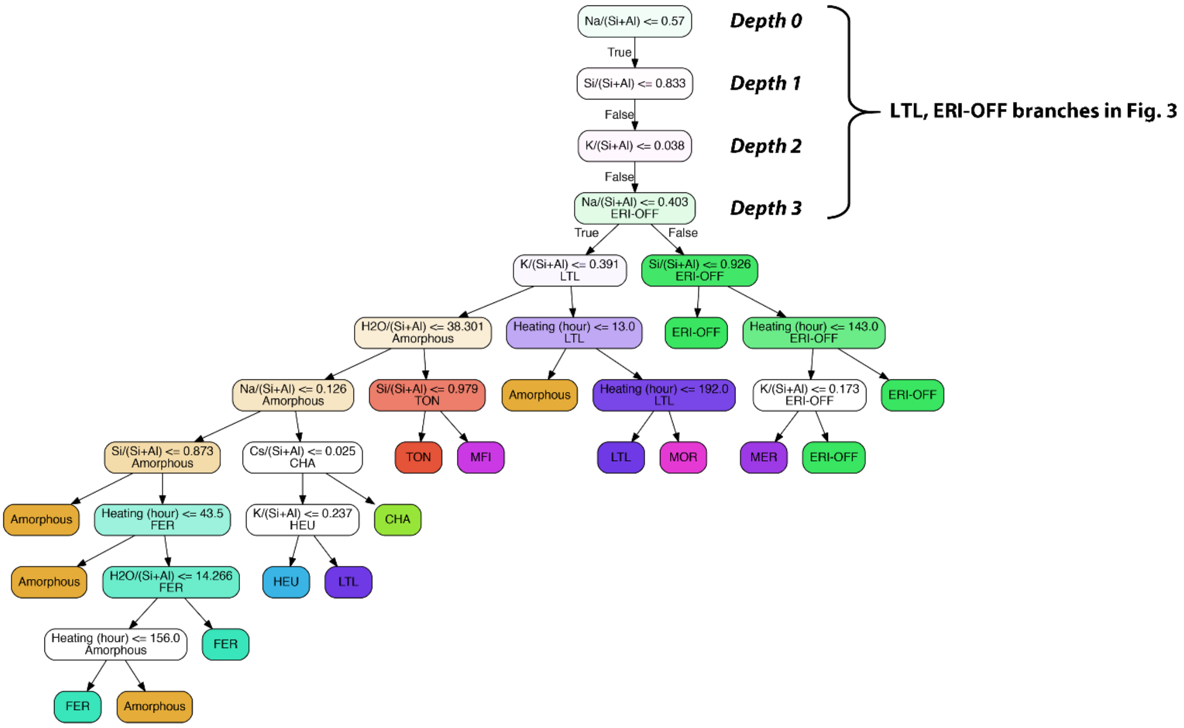

Supplementary Fig. 10 | The complete decision tree beneath the LTL and ERI-OFF branches shown in Fig. 3 in the main article. Below the depth of 4, arrows point left leaves satisfy a decision criterion shown in its root, while those point right do not.

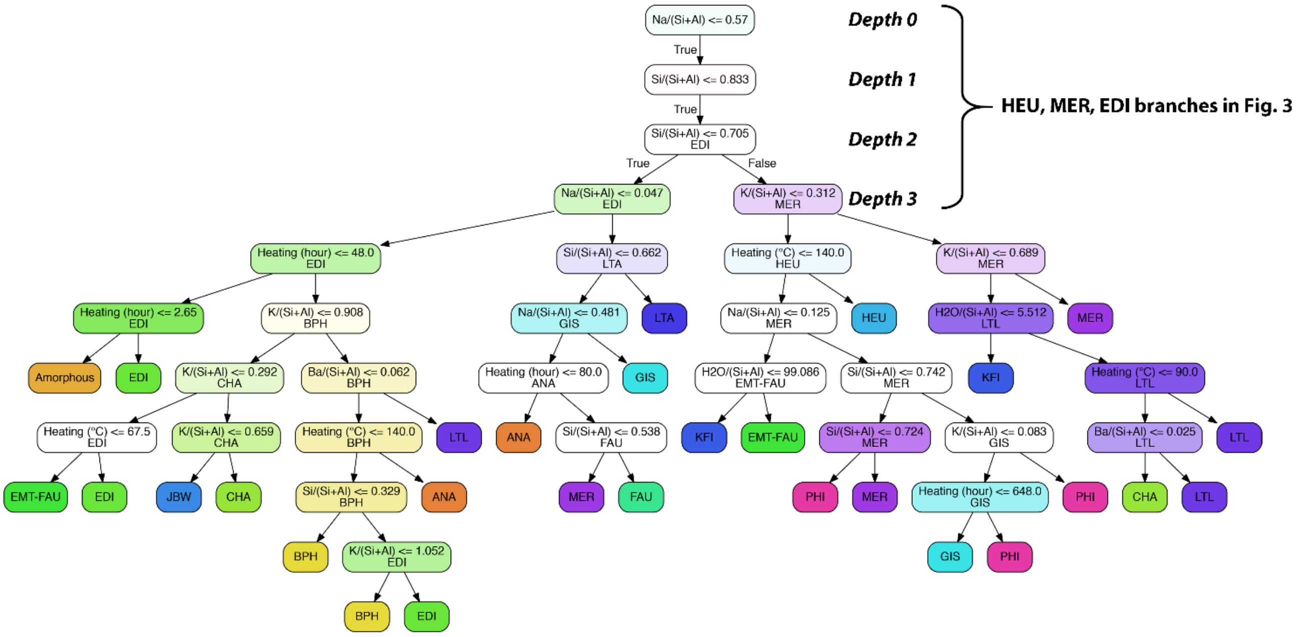

Supplementary Fig. 11 | The complete decision tree beneath the HEU, MER, and EDI branches shown in Fig. 3 in the main article. Below the depth of 4, arrows point left leaves satisfy a decision criterion shown in its root, while those point right do not.

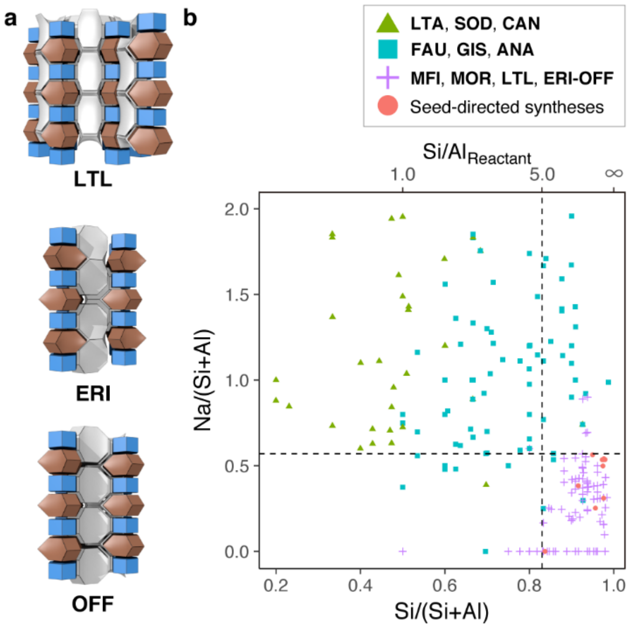

Supplementary Fig. 12 | Structures and synthesizable domains of LTL and ERI-OFF. a, Structural similarity between **LTL**, **ERI**, and **OFF** that share *d6r* and *can* units. **b**, Mapping of synthetic records in the dataset used in the machine learning model and the seed-directed, organic-free syntheses on two synthesis descriptors.

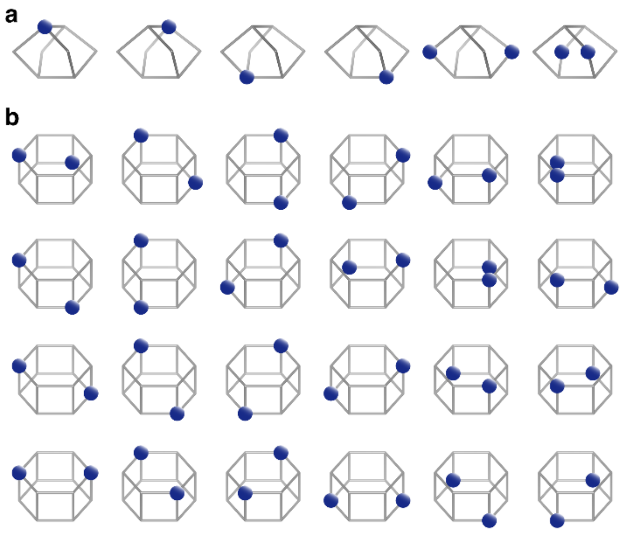

Supplementary Fig. 13 | Aluminum configurations in the building units. a,**b**, All possible configurations of Al in *mor* (**a**) and *d6r* (**b**) units when Al (blue sphere) is introduced as much as possible without forming Al–O–Al and Al–O–Si–O–Al bonds.

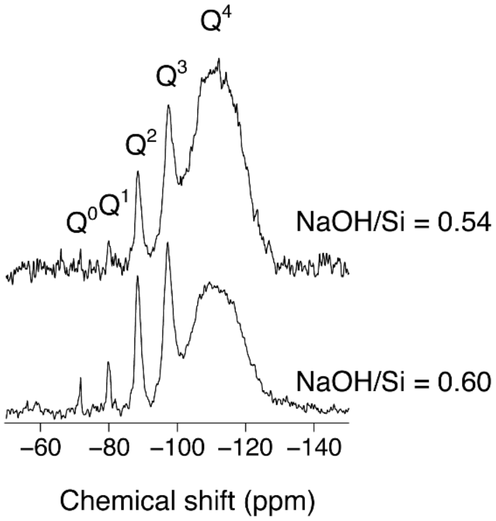

Supplementary Fig. 14 | Solution-state ^{29}Si NMR spectra of transparent sodium silicate solution with different amounts of Na.

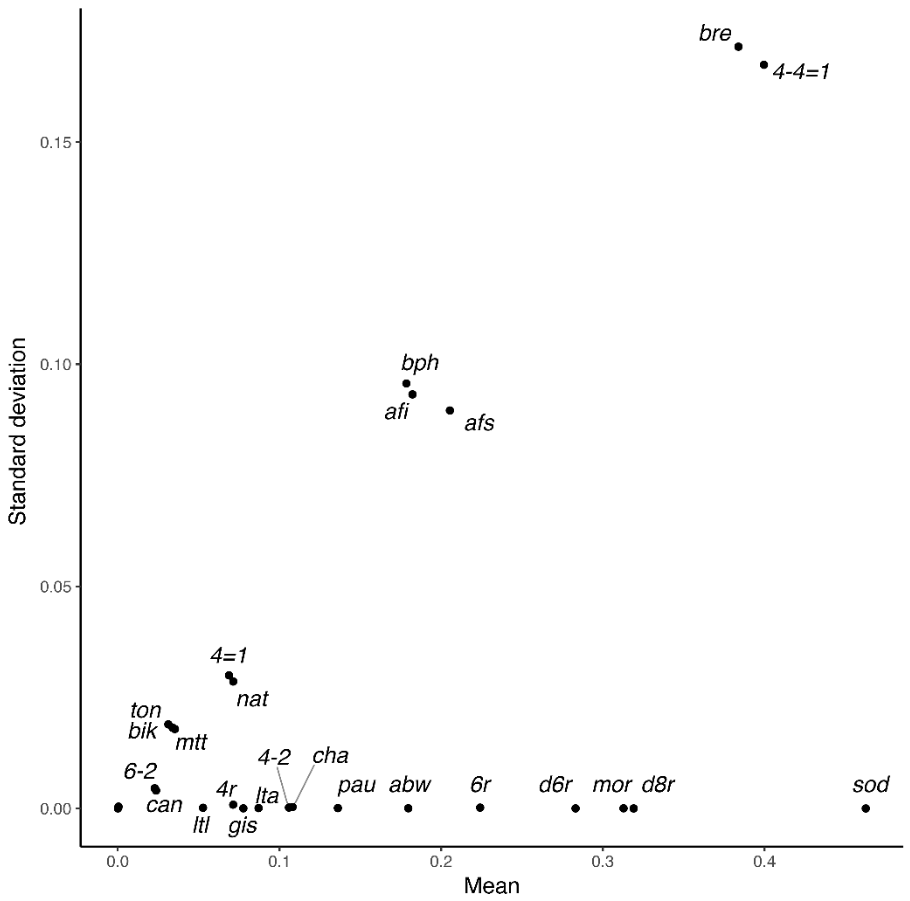

Supplementary Fig. 15 | Standard deviation versus mean of the weights of the structural descriptors calculated from 100 independent numerical optimizations. The building units with low weights are trivial because their presence has less significance in the synthesis–structure relationship. Large standard deviation implies that the structure descriptor is essentially an adjustable parameter with less correlation to the synthesis descriptors.

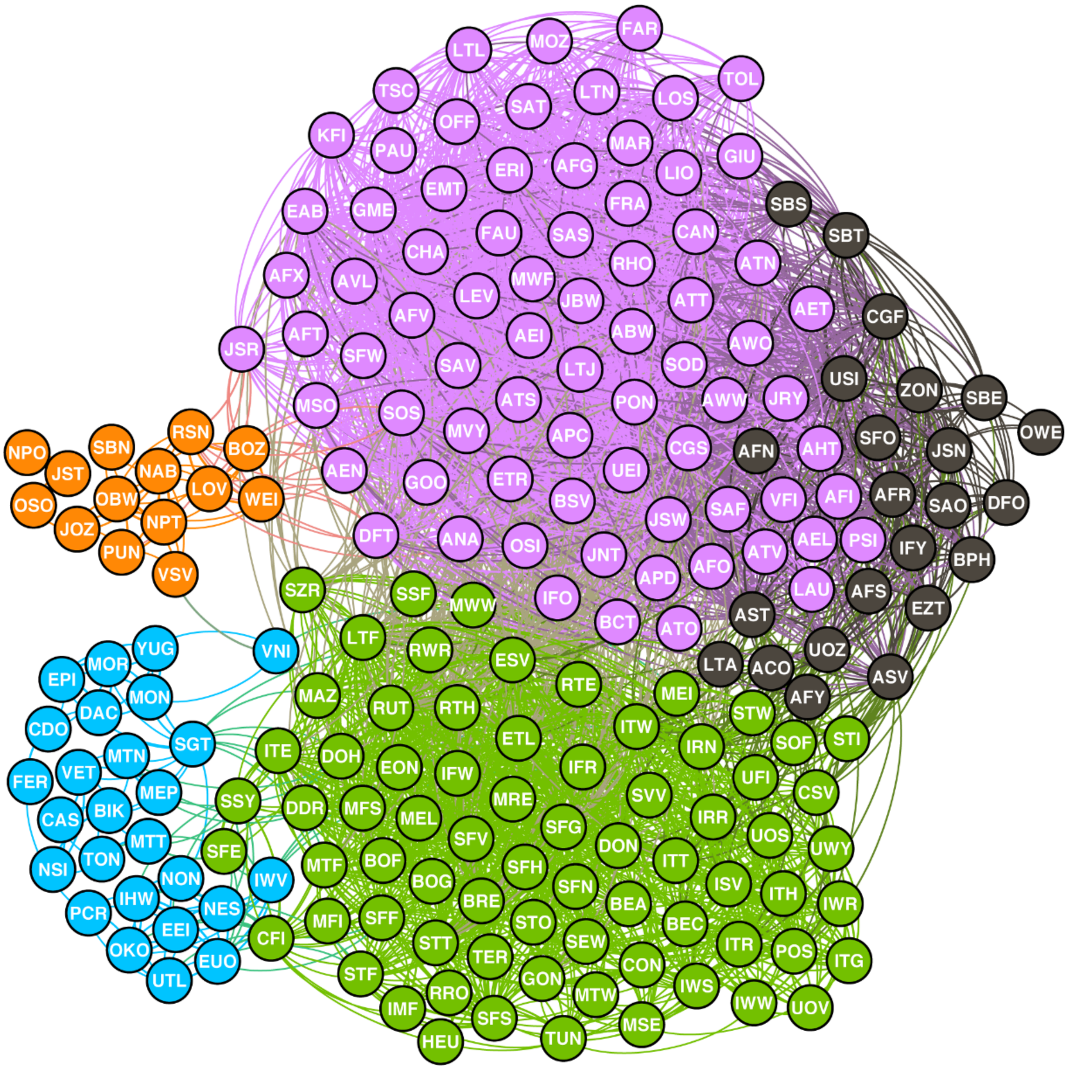

Supplementary Fig. 16 | The structural similarity network constructed based on the assumption that all structure descriptors have equivalent weights (or importance). In contrast to community I in Fig. 5 in the main article, the network constructed without considering the synthesis similarity failed to capture the structural similarities among zeolites, e.g., that between **FAU**, **LTA**, and **SOD**, probably because of the less emphasis of *sod*. Instead, *d4r* was the major driving force to assemble the community for **LTA**, which was negligibly important in the current dataset (Supplementary Fig. 14), but may be important for zeolite syntheses with OSDAs in the presence of germanium and/or fluoride.

Supplementary Table 1 | List of literature used as the data source and reported phases.

Reference	Phase
US Patent 3,760,062 (1973).	**ANA**
J. Cryst. Growth, **294,** 78–82 (2006).	**ANA**
J. Cryst. Growth, **306,** 146–151 (2007).	**ANA**
Catal. Sci. Technol., **4,** 3762–3771 (2014).	**ANA**, **CAN**, **FAU**, **GIS**, **JBW**, **LTA**, **LTL**, **MER**, **MFI**, **MOR**, **SOD**
J. Am. Chem. Soc., **135,** 2641–2652 (2013).	**ANA**, **CAN**, **FAU**, **GIS**, **LTA**, **SOD**
Ing. Eng. Chem. Prod. Res. Dev., **20,** 721–726 (1981).	**ANA**, **MOR**
Stud. Surf. Sci. Catal., **37,** 37–44 (1988).	**ANA**, **LTL**, **MER**, **MFI**, **MOR**
Zeolites, **11,** 116–123 (1991).	**BPH**
US Patent 5,382,420 (1995).	**BPH**
J. Mater. Chem., **15,** 791–797 (2005).	**CAN**
Microporous Mesoporous Mater., **137,** 32–35 (2011).	**CAN**
US Patent 3,030,181 (1962).	**CHA**
J. Phys. Chem., **100,** 4148–4153 (1996).	**CHA**
J. Chem. Soc., Dalton Trans., 1259–1265 (1972).	**CHA**, **LTL**
Zeolites, **15,** 535–539 (1995).	**CHA**, **MER**
US Patent 3,904,738 (1975).	**CHA**, **RHO**
J. Chem. Soc., Dalton Trans., 934–941 (1974).	**EDI**
US Patent 0269472 A1 (2006).	**EDI**
Microporous Mesoporous Mater., **88,** 101–104 (2006).	**EDI**
J. Eur. Ceram. Soc., **26,** 455–458 (2006).	**EDI**
Microporous Mesoporous Mater., **249,** 105–110 (2017).	**EDI**, Amorphous
Verified synthesis of zeolitic materials 2nd (2001).	**EDI**, **FAU**, **LTA**, **LTL**, **MER**, **MOR**, **PHI**
US Patent 3,415,736 (1968).	**EMT-FAU**
US Patent 3,411,874 (1968).	**EMT-FAU**
Chem. Mater., **28,** 4204–4213 (2016).	**EMT-FAU**
Science, **335,** 70–73 (2012).	**EMT-FAU**, **FAU**
Verified synthesis of zeolitic materials 3rd (2016).	**EMT-FAU**, **FAU**
US Patent 2,950,952 (1960).	**ERI-OFF**
US Patent 2,962,355 (1960).	**ERI-OFF**
Stud. Surf. Sci. Catal., **24,** 111–118 (1985).	**ERI-OFF**
Microporous Mesoporous Mater., **41,** 241–251 (2000).	**ERI-OFF**
Microporous Mesoporous Mater., **124,** 117–122 (2009).	**ERI-OFF**
Clays Clay Miner., **59,** 328–335 (2011).	**ERI-OFF**, **MER**, **LTL**
Microporous Mesoporous Mater., **114,** 495–506 (2008).	**ERI-OFF**, **MOR**, Amorphous

US Patent 2,882,244 (1959).	**FAU**
US Patent 3,130,007 (1964).	**FAU**
J. Phys. Chem. B, **108,** 15587–15598 (2004) .	**FAU**
Angew. Chem., Int. Ed., **56,** 13366–13371 (2017).	**FAU**, Amorphous
J. Phys. Chem., **64,** 1567–1571 (1960).	**FAU**, **GIS**, **LTA**, **MER**, **SOD**
Chem. Eur. J., **22,** 16078–16088 (2016).	**FAU**, **GIS**, **MER**, **PHI**
Chem. Mater., **28,** 4906–4916 (2016).	**FAU**, **LTA**, Amorphous
Chem. Eng. Technol., **25,** 273 (2002).	**FER**
Chem. Mater., **18,** 3023–3033 (2006).	**FER**, **MER**, **MOR**, Amorphous
Top. Catal., **52,** 67–74 (2009).	**FER**, **MOR**
Microporous Mesoporous Mater., **158,** 204–208 (2012).	**FER**, **MOR**, Amorphous
US Patent 3,008,803 (1961).	**GIS**
Am. Mineral., **49,** 656 (1964).	**GIS**
J. Mater. Chem., **8,** 233–239 (1998).	**HEU**
Micropor. Mater., **8,** 49–55 (1997).	**HEU**, **MOR**, **PHI**, Amorphous
Microporous Mesoporous Mater., **70,** 63–70 (2004).	**JBW**, **LTA**
US Patent 3,720,753 (1973).	**KFI**, **LTL**
J. Am. Chem. Soc., **131,** 10127–10139 (2009).	**LTA**
Thermochim. Acta, **511,** 37–42 (2010).	**LTA**
Chem. Eur. J., **17,** 6162–6169 (2011).	**LTA**
Chem. Commun., **51,** 269–272 (2015).	**LTA**
Stud. Surf. Sci. Catal., **28,** 177–184 (1986).	**LTA**, **SOD**
Microporous Mesoporous Mater., **76,** 81–89 (2004).	**LTL**
Powder Technol., **145,** 10–19 (2004).	**LTL**
Chem. Mater., **16,** 3381–3389 (2004).	**LTL**
Microporous Mesoporous Mater., **80,** 237–246 (2005).	**LTL**
Monatsh. Chem., **136,** 77–89 (2005).	**LTL**
J. Porous Mater., **14,** 119–126 (2007).	**LTL**
Chem. Eur. J., **17,** 2199–2210 (2011).	**LTL**
Eng. J., **16,** 1–12 (2012).	**LTL**
Chem. Mater., **28,** 1714–1727 (2016).	**LTL**, Amorphous
US Patent 3,012,853 (1961).	**MER**
Microporous Mesoporous Mater., **43,** 61–71 (2001).	**MER**
Clays Clay Miner., **59,** 328–335 (2011).	**MER**
US Patent 4,257,885 (1981).	**MFI**
Zeolites, **7,** 549–553 (1987).	**MFI**
Stud. Surf. Sci. Catal., **28,** 223–230 (1986).	**MFI**, **MOR**, Amorphous
Appl. Catal., A, **181,** 29–38 (1999).	**MOR**, **MFI**, Amorphous
US Patent 4,461,631 (1984).	**MOR**

Microporous Mesoporous Mater., **101,** 57–65 (2007).	**MOR**, Amorphous
Molecular Sieves—II, ACS Symposium Series, 219–232 (1977).	**PHI**
Microporous Mesoporous Mater., **142,** 666–671 (2011).	**SOD**
Zeolites, **14,** 610–619 (1994).	**TON**

Supplementary Table 2 | Test accuracy of machine learning models using heating time, heating temperature, and molar compositions with different standards (i.e., denominators) as synthesis descriptors.

	Standard					
Classifier	Si+Al	Si	Si+Al+M	M	Al	H_2O
XGBoost	0.80	0.79	0.79	0.78	0.78	0.75
Support vector machine	0.66	0.63	0.71	0.69	0.67	0.75
Decision tree	0.76	0.71	0.76	0.69	0.76	0.78
Random forest	0.80	0.80	0.76	0.78	0.80	0.80

Supplementary Table 3 | Test accuracy of machine learning models using heating time, heating temperature, chemical compositions divided by (Si+Al), and additional descriptors encoded as one-hot vectors.

	Additional descriptor				
Classifier	None	Aging	Al source	Si source	Aging, Al and Si sources
XGBoost	0.80	0.80	0.79	0.80	0.79
Support vector machine	0.66	0.69	0.70	0.65	0.71
Decision tree	0.76	0.77	0.76	0.72	0.72
Random forest	0.80	0.79	0.79	0.77	0.82

Supplementary Table 4 | Final compositions of reactants in organic-free, seed-directed synthesis considering structural similarity.

$Si/Al_{Reactant}$	Na/(Si+Al)	Product without seed	Seed	Product with seed	Reference
19	0.56	**MOR**	***BEA**	***BEA**	*J. Phys. Chem. C*, **115,** 744–750 (2011).
17	0.40	**MOR**	**CDO**	**FER**	*J. Mater. Chem.*, **21,** 9494–9497 (2011).
41	0.31	**MFI**	**MFI**	**MTW**	*J. Am. Chem. Soc.*, **134,** 11542–11549 (2012).
36	0.53	**MOR**	**MOR**	**MOR**	*J. Am. Chem. Soc.*, **134,** 11542–11549 (2012).
42	0.54	**MOR**	**FER**	**FER**	*J. Am. Chem. Soc.*, **134,** 11542–11549 (2012).
39	0.54	**MOR**	**MFI**	**MFI**	*J. Am. Chem. Soc.*, **134,** 11542–11549 (2012).
48	0.54	**MOR**	**MEL**	**MEL**	*J. Am. Chem. Soc.*, **134,** 11542–11549 (2012).
5.1	0.00	**MER**	**PAU**	**PAU**	*J. Am. Chem. Soc.*, **134,** 11542–11549 (2012).
11	0.38	**ERI-OFF**	**MAZ**	**MAZ**	*Microporous Mesoporous Mater.*, **186,** 21–28 (2014).
38	0.50	**MOR**	**MSE**	**MSE**	*Chem. Mater.*, **26,** 1250–1259 (2014).
22	0.25	**MFI**	**EUO**	**NES**	*Microporous Mesoporous Mater.*, **215,** 191–198 (2015).

Supplementary Table 5 | Central synthetic condition for each zeolite structure.

							Heating		
Phase	Si [a]	Li [a]	Na [a]	K [a]	Cs [a]	H_2O [a]	(◦C)	(h)	
**ANA**	0.80	–	0.6	–	–	22	165	24	*Ing. Eng. Chem. Prod. Res. Dev.* **17,** 223–227 (1978).
**BPH**	0.14	–	–	4.6	–	137	90	168	*Zeolites* **11,** 116–123 (1991).
**CAN**	0.52	–	1.4	–	–	25	180	504	*Catal. Sci. Technol.* **4,** 3762–3771 (2014).
**CHA**	0.75	–	–	0.62	–	34	80	96	*J. Chem. Soc., Dalton Trans.* 1259–1265 (1972).
**EDI**	0.50	1.5	–	–	–	68	80	24	*J. Eur. Ceram. Soc.* **26,** 455–458 (2006).
**EMT-FAU**	0.88	–	1.6	–	–	11	50	45	*Nat. Mater.* **14,** 447–451 (2015).
**ERI-OFF**	0.93	–	0.49	0.16	–	13	120	168	*Powder Technol.* **206,** 345–352 (2011).
**FAU**	0.71	–	5.7	–	–	140	25	354	*J. Phys. Chem. B* **108,** 15587–15598 (2004).
**FER**	0.91	–	0.23	–	–	32	175	144	*Top. Catal.* **52,** 67–74 (2009).
**GIS**	0.70	–	1.3	–	–	22	100	168	*Chem. Eur. J.* **22,** 16078–16088 (2016).
**HEU**	0.82	–	0.29	–	–	9.9	180	112	*J. Mater. Chem.* **8,** 233–239 (1998).
**JBW**	0.50	–	0.24	–	–	8.6	200	96	*Microporous Mesoporous Mater.* **70,** 63–70 (2004).
**KFI**	0.83	–	–	0.2	0.05	6.4	100	144	US Patent 3,720,753 (1973).
**LTA**	0.50	–	1.5	–	–	26	65	168	*J. Am. Chem. Soc.* **135,** 2641–2652 (2013).
**LTL**	0.86	–	–	0.43	–	21	170	72	*Chem. Mater.* **16,** 3381–3389 (2004).
**MER**	0.71	–	–	0.86	–	23	165	72	*Clays Clay Miner.* **59,** 328–335 (2011).
**MFI**	0.96	–	0.31	–	–	44	190	24	*Stud. Surf. Sci. Catal.* **28,** 223–230 (1986).
**MOR**	0.91	–	–	–	–	27	165	120	*Microporous Mesoporous Mater.* **101,** 57–65 (2007).
**PHI**	0.91	–	0.63	0.32	–	14	100	68	*Molecular Sieves—II*, ACS Symposium Series, 219–232 (1977).
**RHO**	0.83	–	0.45	–	0.05	8.1	100	72	US Patent 3,904,738 (1975).
**SOD**	0.40	–	8	–	–	44	60	52	*Microporous Mesoporous Mater.* **142,** 666–671 (2011).
**TON**	0.97	–	–	0.16	–	48	150	163.2	*Zeolites* **14,** 610–619 (1994).

[a]Divided by (Si+Al).

Supplementary Table 6 | Final compositions of reactants for synthesis of structures in the upper part of community I in Fig. 5 (in the main article) using monoquaternary ammonium cations. Interestingly, they were also within the domain of Na/(Si+Al) ≤ 0.57 and $Si/Al_{Product}$ > 5.0, although such synthesis conditions are not considered in the present dataset. This again suggests the synthesis–structure relationship in this synthesis range.

Phase	$Si/Al_{Reactant}$	Na/(Si+Al)	Organic structure-direction agent	
**AEI**	18	0.51	3,5-Dimethylpiperidinium	*Top. Catal.* **58,** 410–415 (2015).
**AFX**	18	0.09	1,3-Bis(1-adamantyl)imidazolium	*ACS Catal.* **2,** 2490–2495 (2012).
**CHA**	19	0.19	*N,N,N*-Trimethyladamantylammonium	*ACS Catal.* **2,** 2490–2495 (2012).
**EAB**	7.5	0.47	Tetramethylammonium	*J. Chem. Soc. A* 1470–1475 (1970).
**ERI**	30	0.48	Tetramethylammonium	*Stud. Surf. Sci. Catal.* **24,** 105–110 (1985).
**GME**	15	0.50	*cis*-3,5-Dimethylpiperidinium	*Angew. Chem., Int. Ed.* **56,** 13475–13478 (2017).
**LEV**	22	0.19	*N*-Methylquinuclidinium	*ACS Catal.* **2,** 2490–2495 (2012).
**OFF**	10	0.22	Tetramethylammonium	*Chem. Commun.* **49,** 11737–11739 (2013).
**SFW**	13	0.55	*N,N*-Diethyl-5,8-dimethyl-2-azonium bicyclo[3.2.2]nonane	*Chem. Mater.* **28,** 708–711 (2016).

Supplementary Table 7 | Channel systems of structures categorized into the community V in Fig. 5 (in the main article). These multipore systems are often beneficial in catalytic applications because different pore sizes appropriately foster the diffusion of both reactants and products. It is of note that we did not explicitly considered rings larger than *6r* as the building units (structure descriptors) owing to their large degree of freedom.

Phase	Channel system (membered-ring)
**BRE**	8×8
**BOG**	12×10
**CON**	12×10×10
**HEU**	10×8
**ITG**	12×10×10
**IWR**	12×10
**IWW**	12×10×8
**RRO**	10×8
**STI**	10×8
**TER**	10×10
**UOV**	12×10×8

Supplementary Table 8 | Typical synthesis conditions of **EEI**, **EUO**, **NES**, and **IHW**.

Phase	R [a]	R [b]	Si [b]	Na [b]	K [b]	F [b]	H_2O [b]	Heating (◦C)	Heating (h)	Reference
**EEI**	**1**	0.20	0.996	–	0.05	–	41	160	336	*Microporous Mesoporous Mater.* **143**, 6–13 (2011).
**EUO**	**2**	0.22	0.957	0.33	–	–	50	200	22.5	US Patent 4,537,754
**NES**	**3**	0.24	0.952	0.27	–	–	47	180	406	*Nature* **353**, 417–420 (1991).
**IHW**	**4**	0.49	0.980	–	–	0.49	6.9	175	336	*J. Am. Chem. Soc.* **127**, 11560–11561 (2005).

[a]R is organic structure-directing agent. [b]Divided by (Si+Al).

Supplementary Table 9 | Candidates of hyperparameters in support vector machine.

Kernel	RBF, Linear	2 candidates
C	$2^{-15}, 2^{-14}, \ldots, 2^{5}$	21 candidates
γ	$2^{-14}, \ldots, 2^{3}$	18 candidates

Supplementary Table 10 | Candidates of hyperparameters in random forest.

Number of trees	10, 50, 100, 300, 1000	5 candidates

Supplementary Table 11 | Candidates of hyperparameters in decision tree.

Criterion of split	Gini impurity, Information gain	2 candidates
Minimum number of samples required to split	2, 10, 20	3 candidates
Maximum depth of the tree	Not specified, 2, 5, 10	4 candidates
Minimum number of samples required to be at a leaf node	1, 5, 10	3 candidates
Maximum number of leaf nodes	Not specified, 5, 10, 20	4 candidates

Supplementary Table 12 | Candidates of hyperparameters in XGBoost.

Learning rate	10^{-3}–0.5	Continuous
Minimum sum of instance weight	0.5, 1, 2	3 candidates
Maximum depth of a tree	1, 2, 3, … , 12	12 candidates
Minimum loss reduction	0–0.5	Continuous
Maximum delta step	0, 1, 2	3 candidates
Subsample ratio of the training instances	0.5−1	Continuous
Subsample ratio of columns	0.5−1	Continuous
